# Supplementary material for: An organ-on-chip model of pulmonary arterial hypertension identifies a BMPR2-SOX17-prostacyclin signalling axis
Source: Commun Biol. 2022 Nov 7;5:1192. doi: 10.1038/s42003-022-04169-z (PMC9640600; doi:10.1038/s42003-022-04169-z)
Supplement: Supplementary file 2 — Supplementary Information [file 42003_2022_4169_MOESM2_ESM.pdf]

SUPPLEMENTARY INFORMATION

AN ORGAN-ON-CHIP MODEL OF PULMONARY ARTERIAL HYPERTENSION IDENTIFIES A BMPR2-SOX17-PROSTACYCLIN SIGNALLING AXIS by Alexander Ainscough et al.

SUPPLEMENTARY METHODS

Photolithography

Individual photomasks were designed for Endothelial and Smooth Muscle Cell chambers using AutoCAD 2017 (Autodesk, CA, USA) and printed on high resolution photomask films (Micro Lithography Services Ltd, UK). Photolithography, soft lithography and pulmonary artery-on-a-chip fabrication were all conducted in an ISO Class 5 Cleanroom (<100,000 particles/m^3). The fabrication procedure was as follows: 100 mm diameter single side polished silicon dioxide wafers with resistivity of 1-10 Ω/cm and thickness of 525 μm (Inseto, UK) were sequentially cleaned in isopropanol, acetone and isopropanol again before drying under a gentle stream of N_2 gas followed by dehydration by hotplate heating at 95°C. SU-8 2100 (A-Gas, UK) was spin coated onto the polished surface of the wafer at 1500rpm, corresponding to a 200 μm feature height. Wafers were pre-exposure baked on hotplates for 5 minutes at 65°C and 12 minutes at 95°C before equilibrating to room temperature. The SU-8 coated wafer was placed inside a UV exposure unit (OAI, CA, USA) containing a 360 nm long pass filter (RS Laser Components, UK) and the appropriate photomask prior to 260 mJ/cm^2 UV light exposure. Wafers were baked post-exposure at 65°C for 5 minutes and 95°C for 12 minutes on hotplates before developing in Microposit EC Solvent (A-Gas, UK). Developed wafers were placed inside a desiccator along with 35μl of Trichloro(1H,1H,2H,2H-perfluorooctyl)silane (Cat: 448931; Sigma, UK) deposited on a fresh microscope slide. The silane was vaporised upon evacuation of the desiccator under vacuum conditions and was left to deposit on the wafer surface for 1 hour.

Soft lithography

PDMS (Sylgard 184 Elastomer Kit, Cat: 634165S, Dow Corning, USA) was weighed and mixed at a ratio of 10:1 (base to curing agent) and poured onto SU-8 master moulds. 4 mm uncured PDMS was poured onto the negative impressions of the SU-8 patterned wafers for the upper (endothelial) compartments and 1 mm was poured onto SU-8 patterned wafers for the lower (smooth muscle) compartments to enable downstream optical imaging. The mixture was degassed in a desiccator for 1 hour and cured on hot plates for 2 hours at 65°C. PDMS chips were excised using a disposable scalpel, cut to size using a single edge razor blade and had access ports punched using a 0.75 mm biopsy punch (Cat: 504529, World Precision Instruments, Hertfordshire, UK).

Pulmonary artery-on-a-chip fabrication

PDMS chips were plasma activated/sterilised for 60 seconds in a plasma cleaner (Harrick Plasma, NY, USA) and immediately immersed in a solution of anhydrous ethanol containing 2.5% GLYMO (Cat: 440167; Sigma Aldrich, Dorset, UK) for 20 minutes at room temperature. Porous PET membranes (0.4 μm pores; $2x10^6$ pores/cm^2; it4ip, Louvain-la-Neuve, Belgium) were plasma activated/sterilised for 60 seconds, immersed in a solution of anhydrous ethanol containing 10% APTES (Cat: 440140; Sigma Aldrich, Dorset, UK) and heated at 65°C for 20 minutes. Chips and membranes were rinsed in IPA, dried in a stream of N2 gas, manually aligned and brought into conformal contact. Chip bonding was completed after overnight curing at 100°C in a convection oven. 20G stainless steel tubes with a length of 1.5 cm (Coopers Needle Works, UK) were carefully bent to 90° angles using pliers, inserted into access ports and secured using two-part epoxy glue (Araldite, USA).

HPAEC and HPASMC co-culture in Transwell filters

12 mm Transwell inserts containing a polyethylene terephthalate (PET) membrane with 0.4 μm pores and a pore density of (2.0 ± 0.4) X 10^6 pores per cm^2 (Cat no: 353180, Corning, USA) were coated with sterile-filtered 100 μg/mL rat tail collagen (BD Biosciences, UK; Cat: 354236) in 20 mM acetic acid (Honeywell, UK; Cat no: 695092) for 1 hour at room temperature. First, the Transwell inserts

were inverted, with the bottom part of the filter facing up and 125,000 HPASMCs suspended in 100 μL of co-culture medium were seeded onto the surface of the filter. After 1 hour, the unattached cells were gently removed with PBS and the inserts were reverted upright and placed in the wells of a 12-well plate filled with 0.5 mL co-culture medium, with HPASMCs facing down. Then, 125,000 HPAECs suspended in 0.3 mL of co-culture medium were plated inside the insert, to create a direct endothelial-smooth muscle cell co-culture on both sides of the PET membrane. Culture media in the top and bottom compartment of the Transwell dish were changed every 48 hours. Cells were used for experiments 2 days post-confluence.

RNA Isolation

Cultured cells wells were washed in PBS and trypsinised (Cat: 25200072; Gibco, UK) to facilitate cell detachment. Trypsin was neutralised with DMEM containing 10% FBS and the cell suspension was collected in RNase-free LoBind 1.5 mL microcentrifuge tubes (Cat: Z666548-250EA; Merck Life Science, UK). Cells were centrifuged for 5 minutes at 5000 *g* at room temperature and residual medium was aspirated without disturbing the cell pellet. Tubes were either stored at -80°C or taken immediately for RNA isolation. Before the RNA isolation, all surfaces and pipettes were treated with RNase-Zap RNase Decontamination Solution (Cat: AM9780; Invitrogen, UK) and sprayed with 70% Ethanol. Total RNA was extracted using either a Monarch Total RNA Miniprep Kit (Cat: T2010S; New England Biolabs, MA, USA) or RNeasy Plus Micro Kit (Cat: 74034; Qiagen, Germany), according to manufacturer's protocols, with the modification of 0.5-fold reduction in the recommended centrifugation speed in all steps except for the final wash and elution in RNase-free water. Double elutions were performed to maximise RNA recovery from spin columns. Total RNA levels were quantified using a NanoDrop™ ND2000 spectrophotometer (Thermo Scientific, UK). The ratio of absorbance at 260 nm and 280 nm (A260/A280) was used to evaluate RNA purity, with values above 1.8 considered acceptable quality. RNA was then stored at -80°C for future experiments.

Reverse Transcription

50 ng (from chips) or 100 ng (from all other samples) of extracted RNA was reverse transcribed into cDNA using a LunaScript RT SuperMix Kit (Cat: E3010L; New England Biolabs, MA, USA). Samples were prepared on ice for RT in PCR-certified 96 well plates in a total volume of 20 µL. cDNA samples were all diluted with RNase-free water to make a 1 ng/µL cDNA solution (30 µL or 70 µL was added to chip and all other sample cDNAs respectively) and either stored at -20°C or immediately used for qPCR. Components of RT reaction mix and RT cycle are detailed in **Supplementary Tables 5 and 6**.

qPCR

1 µL of 1 ng cDNA was used in each individual well for PCR. Master mixes for each gene of interest were created using Luna Universal qPCR Master Mix (Cat: M3003E; New England Biolabs, MA, USA), primers and water, as outlined in **Supplementary Table 7**. All primer sequences were designed from FASTA sequences, where at least one primer pair spanned an exon-exon junction (PubMed, NCBI). The relative expression of a gene was determined using the 2-ΔΔCt method, with target genes normalised to glyceraldehyde 3-phosphate dehydrogenase (GAPDH) expression. A list of primers is shown in **Supplementary Table 8**.

Proteomic analysis

Protein centration of HPAECs samples was determined by Bradford assay and protein was digested for MS analysis by single-pot digestion. In 660 µL of 100 mM ammonium bicarbonate, 20 µg of protein was reduced with 10 mM tris(2-carboxyehtyl)phosphene), alkylated with 40 mM chloracetamide and digested with 0.2 µg of sequencing grade modified trypsin (Promega) at 37°C for 16 hours. Digestion was halted by the addition of TFA to 0.5% (v/v) and immediate desalting. Desalting was performed using HLB uElution plates (Waters) with a vacuum manifold. The plate was conditioned three times with 100 µL of elution buffer (80% acetonitrile, 0.05% formic acid) and equilibrated three times with 100 µL of wash buffer (0.05% formic acid). Samples were passed slowly through the HLB plate and washed 3 times with 100 µL of wash buffer, ensuring the bed was

completely empty between each wash. Desalted peptides were eluted with 3 x 50 µL elution buffer and dried at 45°C in a vacuum centrifuge. Samples were reconstituted in 40 µL 0.1% FA and a peptide assay was performed. Subsequently, samples were adjusted to 0.1 µg/µL for LC-MS and 2 µL was injected onto a 180 µm x 20 mm C18 trap column (NanoEase M/Z Symmetry, Waters) for 3 minutes at 15 µLper minute in 5% acetonitrile, 0.1% formic acid. Peptides were separated on a 75 µm x 200 mm C18 column (nanoEase M/Z HSS T3, Waters) with a 90-minute gradient of 5-35% acetonitrile with 0.1% formic acid at 0.3 µL/minute on a Acquity M-Class UPLC (Waters) interfaced with a Synapt G2S mass spectrometer (Waters). Data was acquired in positive resolution mode, using HDMSE over 50-2000mz with a scan time of 0.5 seconds with lockmass acquired for 3 scans every 1 minute (Leucine-enkephalin). For high-energy scans, the collision energy was ramped between 19-45V with a cone voltage of 40V. Samples were randomised and each sample was interspaced with a blank to prevent carry over.

Data was processed using Progenesis QI for Proteomics with lock mass correction (556.2771 Da), with peak picking performed with default settings, but maximum charge set to 6. Data was searching using a human reference proteome (Swissprot, downloaded on 28/06/2021) with trypsin digestion and up to 2 missed cleavages specified. Methionine oxidation was set to variable and cysteine carbamidomethylation set as a fixed modification. FDR filtering was performed to 4%, with 3 fragments per peptide, 5 fragments per protein and 1 peptide per protein.

Identification of SOX17 binding sites

The promoter region coordinates of the genes investigated were chosen based on the GeneHancer Identifiers[1] provided in Genecards [2] (**Supplementary Table 9**). For Dickkopf WNT Signaling Pathway Inhibitor 2 (*DKK2*) the promoter sequences were chosen largely based on Histone 3 Lysine 4 trimethylation (H3K4me3) marks from HUVECs since the sequences from the GeneHancer Identifiers did not cover a sufficient length of the expected promoter region. These genomic

coordinates were then submitted to the UCSC Genome Browser on Human GRCh38/hg38 build [3] in order to retrieve the sequence in these locations. In this session the following tracks were added: Gencode v39[3], NCBI refseq track[4], which provides gene annotations, ENCODE candidate cis-regulatory elements (cCREs)[5], ENCODE DNase I hypersensitivity from HUVECs[6] and HUVEC Histone 3 Lysine 24 acetylation (H3K27ac) and Histone 3 Lysine 4 mono- and tri-methylation (H3K4me1 & H3K4me3) data from the Blueprint project[7]. The sequences of putative enhancers of the genes investigated (**Supplementary Table 10**) were chosen based on the integration of H3K27Ac and H3K4me1 marks, which indicate active regulatory regions and enhancer regions respectively. The sequences were then submitted to the Catalog of Inferred Sequence Binding Preferences (CIS-BP) library[8] and were examined for potential SOX17 binding motifs.

Permeability assays

Transwell assay:

500 μL medium containing 1 mg/mL FITC-Dextran [9] was added to the apical compartment of the transwell insert and was incubated for 1 hour. 1 mL from the lower compartment was collected after one hour and fluorescence of FITC-dextran was measured in a GLOMAX spectrophotometer (Promega, USA) at excitation/emission 490/525 nm. For thrombin-based experiments, 1 U/mL thrombin (T7513, Sigma Aldrich, Dorset, UK) was added to the co-culture media containing 1 mg/mL FITC-Dextran and the experiment was performed as described above.

Microfluidic permeability assay**:**

1 mg/mL 40kDa FITC-Dextran (FD40S, Sigma Aldrich, Dorset, UK) was added to co-culture medium and perfused through the top channel at 6 dynes/cm^2. Perfusion was stopped after one hour and the lower smooth muscle cell channel was gently flushed with 250 μL of medium and collected for analysis on a GLOMAX spectrophotometer (Promega, USA) at excitation/emission 490/525 nm. Apparent permeability Papp [cm/s] was determined using the formula[10]:

$P_{app} = V_r \cdot C_r / [A \cdot t \cdot (C_{d-out} \cdot V_d + C_r \cdot V_r) / (V_d + V_r)]$

Where:

- V_r is volume of receiving channel at time *t*
- V_d is volume of dosing channel at time *t*;
- A is area of membrane, which for our system is 0.1433333cm^2
- C_r is measured concentration of tracer in the receiving channel
- $C_{d\text{-}out}$ is measured concentration of tracer in the dosing channel effluent

Immunostaining

Cells were fixed in 4% paraformaldehyde (Sigma Aldrich, UK) (w/v) in PBS for 20 minutes at room temperature, washed 3x in PBS and permeabilised in 0.1% (v/v) Triton-X-100 (Sigma Aldrich, UK) in PBS for 5 minutes. Samples were then incubated in blocking buffer containing: 0.1% Triton-X-100 (Sigma Aldrich, UK) (v/v) in PBS with either 5% (v/v) Normal Goat Serum (NGS; Cat: 31873, Invitrogen, UK) or 2% (v/v) BSA (A9418, Sigma Aldrich, UK) in PBS for 30 minutes at room temperature. Primary antibodies were added at appropriate dilutions in a solution containing 2.5% (v/v) NGS and 0.05% (v/v) Triton-X-100 in PBS for 2 hours at room temperature or left overnight at 4°C. Samples were washed 3x in PBS and secondary antibodies were incubated accordingly for 1 hour. Samples were washed 3x in PBS and were mounted in VECTASHIELD® Antifade Mounting Medium with DAPI (Vector Laboratories, UK, Cat: H-1200). A list of primary and secondary antibodies used is shown in **Supplementary Table 11**. Immunostained samples were visualised under a Zeiss LSM-780 inverted confocal laser scanning microscope (Carl Zeiss AG, Germany) or a fluorescent Zeiss AxioObserver widefield microscope (Carl Zeiss AG, Germany) with x5, x10 or x20 objective.

Histological sections of lung tissues from treatment-naïve PAH patients at lung transplantation (n=2), and control tissues comprising uninvolved regions of lobectomy specimens from 2 unused donor lungs were from the tissue archives at Hammersmith Hospital, Imperial College London. Tissue samples were fixed in 10% (v/v) formaldehyde in PBS and embedded in wax, and sections were processed for immunohistochemistry as previously described[11]. Briefly, dewaxed and deparaffinised

tissue sections were blocked for 1 hour at room temperature with 3% (v/v) normal horse serum (Vector Laboratories) in 1X PBS containing 0.1% (w/v) bovine serum albumin (Sigma-Aldrich) and 0.01% (v/v) sodium azide (Sigma-Aldrich) and then incubated with anti- von Willebrand Factor antibody (to label endothelium) and anti- smooth muscle actin antibody (to label smooth muscle cells) at 4°C overnight. After 3x washes in PBS, slides were incubated with secondary, fluorescently-labelled anti-rabbit and anti-mouse antibodies for 30 minutes at room temperature. Following immunostaining, tissues were mounted in VECTASHIELD® Antifade Mounting Medium with DAPI (Vector Laboratories, UK, Cat: H-1200) and examined under a fluorescent confocal microscope (Leica, TCS SP5, Leica Biosystems, Bretton, Peterborough).
List of primary and secondary antibodies used for immunostaining is provided in **Supplementary Table 11**.

EdU Proliferation Assay

Cell proliferation was quantified using an EdU Cell Proliferation Assay Kit (EdU-594, EMD Millipore Corp, USA, Cat. No. 17-10527). EdU nucleotides were added directly to cell culture medium at a dilution of 1:1000, 24 hours before the end of experiment. Cells were fixed, permeabilized and blocked as in immunostaining method, and were treated with the assay reaction cocktail, prepared according to the manufacturer's instructions. EdU positive cells were visualized under a fluorescent Zeiss AxioObserver widefield microscope (Carl Zeiss AG, Germany). Images and z-stacks were taken with x10 objective and were analysed with ImageJ software. Data is presented as a percentage of the number of EdU positive cells vs the total cell number.

Hexokinase activity measurement.

Hexokinase activity was measured in pulled samples of PASMCs (1.5 $x10^5$ cells/treatment) under noromoxic, hypoxic and "double hit" conditions with Hexokinase Assay Kit (Colorimentric) (Abcam, ab136957), according to the manufacturer's protocol (https://www.abcam.com/ps/products/136/ab136957/documents/Hexokinase-Assay-Kit-protocol-book-v7a-ab136957%20(website).pdf). In the Hexokinase Assay kit, glucose is converted to glucose-

6-phosphate by hexokinase; the glucose-6-phosphate is oxidized by glucose-6-phosphate dehydrogenase to form NADH, which reduces a colorless probe to a colored product with strong absorbance at 450 nm.

Drug treatment

Imatinib Mesylate (Enzo life sciences, UK; Cat: ALX-270-492-M025) and Ambrisentan (AstraZeneca) were added directly to cell culture medium at clinically relevant concentrations of 10 µM[12] and 1.25 nM[13], respectively. AZD5153 (AstraZeneca) was added at the concentration of 16.5 nM, based on pre-clinical studies with BET inhibitors[14] and the *in vitro* dose-response experiments. Cells were incubated with the inhibitors for 24 hours under normoxic (21% O_2) or hypoxic (2% O_2) conditions accordingly. 4.2 mM stock solution of AZD5153 in DMSO was diluted to the final concentration of 16.5 nM and equivalent amount of DMSO was added to vehicle controls. Similarly, 50 mM stock solution of Ambrisentan in DMSO was diluted to the final concentation of 1.25 nM, with appropriate vehicle controls.

Apoptosis assay

Apoptosis was measured using a Click-iT Plus TUNEL Assay for In Situ Apoptosis Detection Kit with Alexa Fluor 647 dye (Life Technologies, Cat: C10619), as per the manufacturer's instructions. TUNEL positive cells were visualized under a fluorescent Zeiss AxioObserver widefield microscope (Carl Zeiss AG, Germany). Images and z-stacks were taken under x10 objective and analysed with ImageJ software. Data is presented as a percentage of the number of TUNEL positive cells vs total cell number.

Western blotting

Cells were washed 3x in ice-cold PBS and lysed using RIPA buffer (Sigma, UK) containing 1x proteinase and phosphatase inhibitor cocktail (ThermoFisher, UK; cat no: 78440). Protein concentration in samples was measured using a Pierce™ BCA Protein Assay Kit (Thermo Fisher Scientific, UK, 23225) using standard protocols and absorbance was measured in a GLOMAX

spectrophotometer (Glomax™ luminometer; Promega, USA). Samples were resolved with 4x Laemmli SDS reducing sample buffer (Alfa Aesar, MA, USA; Cat No: J60015) and heated at 95°C for 10 minutes. 10-20 µg of protein per sample was loaded onto either 10% Mini-PROTEAN® TGX™ Precast Protein Gels (10-well, 50 µL; Cat No: #4561034; Bio-Rad, CA, USA) or a pre-cast 4-12% bis-tris Nu-PAGE gel (Invitrogen, UK). 5 µL of Spectra Multicolour Broad Range Protein Ladder (ThermoFisher, UK; cat no: 26634) was loaded into the first well of each gel in order to estimate protein molecular weights within samples during blot imaging. Samples were separated by SDS-PAGE in NuPAGE™ MES (2-(N-morpholino)ethanesulfonic acid; Cat: NP0002; Invitrogen, UK) or NuPAGE™ MOPS (3-(N-morpholino)propanesulfonic acid; Cat: NP0001; Invitrogen, UK) buffer at 200V for 35 minutes. Proteins were transferred onto PVDF or nitrocellulose membranes using a trans-blot turbo transfer system (Biorad, UK). Membranes were blocked in freshly prepared 5% (w/v) Semi-skimmed milk (Cat: 70166-500G; Merck, UK) or 5% (w/v) bovine serum albumin (BSA) (Cat: A9418; Sigma, UK) in TBST (2.42g Tris, 8g Sodium Chloride, 1L double distilled Millipore water, 1ml Tween-20, adjusted to pH 7.8) for one hour and then incubated overnight at 4°C with the appropriate primary antibody. After that, membranes were washed 5x for 3 minutes each with fresh TBST and incubated with appropriate HRP-conjugated secondary antibodies in TBST for one hour and then washed 3x 15 minutes in TBST. Bands were visualised using Crescendo ECL detection reagent (Merck-Millipore, USA) with a ChemiDoc MP Imaging System (Biorad, UK). Primary and secondary antibodies used for western blotting are listed in **Supplementary Table 12**. Band intensity was determined by densitometry using ImageJ software. Expression of proteins of interest was normalised to β-actin.

Statistical analysis.

All graphs were either plotted in RStudio or Graphpad Prism 8 software (Graphpad Software Inc, CA, USA), with statistical tests performed, as appropriate. In bar graphs, error bars indicate the standard error of the mean (SEM). When comparing two sample groups, normally distributed data was

analysed using an unpaired student’s t test. For comparisons of two or more sample groups, one or two way ANOVA was used, as appropriate. The threshold for statistical significance was $P < 0.05$.

Simulation of PA-on-a-chip

Finite Element Method-based simulations were used to simulate the 3D fluid structure interaction within the PAOC device using COMSOL Multiphysics version 4.4. PDMS walls model parameters are shown in Supplementary Table 1 and a schematic of the model is shown in figure M1.

Supplementary Table 1. Rigid PDMS walls model parameters

Th	10[um]	Membrane thickness
W	1[mm]	channel thickness
H_{EC}	200[um]	Rest endothelial channel height
L_{un}	1[cm]	artery on a chip union length
L_{arm}	0.5[cm]	channel arm length
H_{SMC}	200[um]	Rest smooth muscle cell channel height
D_{pin}	1[mm]	steel connector diameter
D_{con}	1.1[mm]	entry/exit connector port diameter
Q_{EC}	0.35[ml/min]	endothelial cell channel volumetric flux
Q_{SMC}	10[ul/h]	smooth muscle channel volumetric flux
P_{EC_out}	See Equation S1	outlet pressure endothelial channel
P_{SMC_out}	See Equation S1	outlet pressure smooth muscle channel
E_{pdms}	1[MPa]	PDMS Young's Modulus
nu_{pdms}	0.499	PDMS Poisson's ratio
θ	pi/4[rad]	SMC channel arm rotation angle theta
td	1[mm]	tube diameter
tl	160[cm]	tube length
mu_{w25}	8.9E-4[Pa*s]	dynamic viscosity of water
rho_{w25}	0.997[g/cm^3]	density of water
E_{pet}	2950[MPa]	PET Young's modulus. E_{PET} values from databases were 2.67, 2.95, 2.985, 3 and 3.15 GPa. The average value was used.[1-5]
rho_{pet}	1250[kg/m^3]	density of PET. The density of PET ranges from 1100 - 1400kgm^{-3}, thus the average value of 1250kgm^{-3} was chosen.[1-5]
nu_{pet}	0.34	PET Poisson's ratio. The Poisson's ratio varies from 0.33-0.35and thus 0.34 was chosen as the average value.

Rigid PDMS wall simulation setup

Figure M1. *Geometry and boundary conditions of Rigid PDMS wall model. a) key dimensions of the microchannels and PET membrane; channel width 'W', channel length 'L', coculture region length 'L_{un}', channel arm length 'L_{arm}', smooth muscle channel arm bend angle 'θ', membrane thickness*

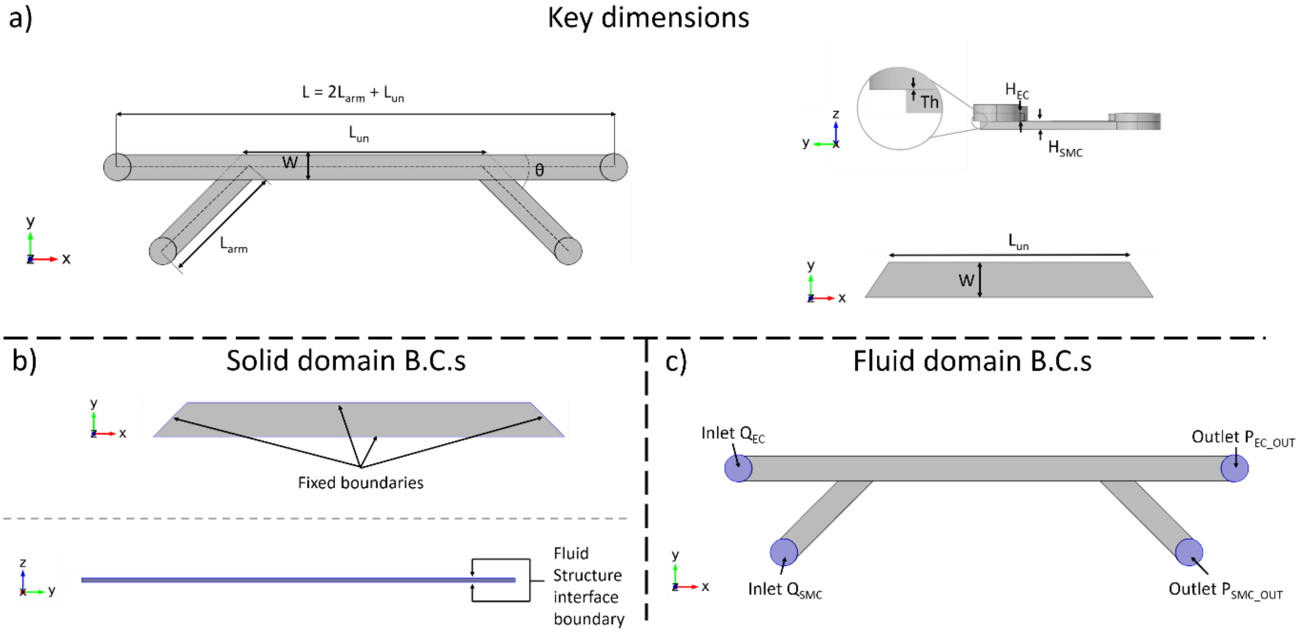

'Th', and channel heights for the endothelial and smooth muscle channels 'H_{EC}' and 'H_{SMC}' respectively. b) PET membrane domain boundary conditions; fixed (top) and Fluid-Structure Interface boundaries. c) Fluid domain boundary conditions; Volumetric flow rates Q_{EC} and Q_{SMC} are applied at the inlets of the endothelial and smooth muscle channels respectively. Outlet pressures P_{EC_OUT} and P_{SMC_OUT} are applied at the outlets of the endothelial and smooth muscle channel respectively.

The rigid PDMS wall model geometry is composed of three domains: one solid and two fluid. These correspond with the PET membrane and the two fluid channels. The PET membrane was modelled as a linear Elastic material with Properties listed in **Supplementary Table 1**. Whereas the fluid domain was modelled as water whose properties are given in **Supplementary Table 1**. The solid (PET membrane) surfaces not in conformal contact with the fluid domains were fixed. The final two faces of the PET membrane were given a fluid structure interface boundary condition.

Q_{EC} and Q_{SMC} are the volumetric flow rates at the Endothelial channel inlet and Smooth muscle cell channel inlet, respectively. At the outlets of each channel, the Hagen-Poiseuille equation shown in equation S1 is applied. Q is replaced by Q_{EC} in the EC channel and Q_{SMC} in the SMC channel. L_{tube} and D_{tube} for both channels are replaced by tl and td respectively. Lastly, the no slip boundary condition is applied to rigid walls in the channel (non-PET membrane walls).

$$P_{out} = \frac{128\mu Q L_{tube}}{\pi {D_{tube}}^4}$$

Equation S1 – Hagen-Poiseuille equation to obtain outlet pressure P_{out}.

The final boundary condition is the fluid structure interface boundary in which all four equations below need to be fulfilled. Where 'σ' is the stress tensor of the solid, 'n' is the vector normal to the wall, 'Γ' is the stress tensor of the fluid, 'u_f' is the fluid velocity vector, 'u_w' is the wall velocity vector, 'u_s' is the deformation of the solid, 't' is time, and 'u_m' is the deformation of mesh.

$$\sigma \cdot n = \Gamma \cdot n \qquad u_f = u_w \qquad u_w = \frac{\partial u_s}{\partial t} \qquad \frac{\partial u_s}{\partial t} = \frac{\partial u_m}{\partial t}$$

A custom meshing sequence was applied to the model with 4524195 degrees of freedom. The Simulation time was 5 hours 30 min 8 sec and required 31.44 GB physical memory and 56.89 GB virtual memory.

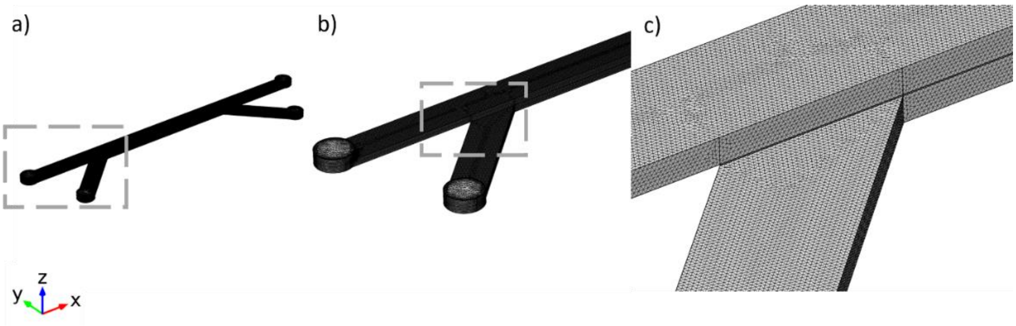

Figure M2. *Tetrahedral mesh in rigid PDMS walls model. a) Full model elements are too fine to see. b) 2x zoom reveals some of the coarser elements at the inlets c) Tetrahedral mesh*

Source databases used in model simulation:

Material Properties Database. http://matweb.com/

Polymer Properties Database https://polymerdatabase.com/polymer%20classes/Intro.html

Material Properties Database https://designerdata.nl/

Plastics and Elastomers Properties Database https://omnexus.specialchem.com/

Polymer Mechanical Properties Database
http://www.goodfellow.com/catalogue/GFCat2C.php?ewd_token=oGMPLDtnz3n13gndyuSlwIkTl0W9Ui&n=yxx30n0WFpfmeOxK6s9CprjvNbsCsC&ewd_urlNo=GFCat26&type=30&prop=3

SUPPLEMENTARY TABLES

Supplementary Table 2. Human pulmonary artery endothelial and smooth muscle cells used in experiments. The table indicates donors, age, gender, and population doubling time. All donors were non-smokers.

**Lot #**	**Age**	**Gender**	**Doubling Time**
**HPAECs (Promocell)**			
**431Z031**	23	Female	48.3 h
**433Z034**	34	Female	23.6 h
**458Z016.12**	51	Female	28.5 h
**451Z031.14**	68	Male	25.4 h
**HPASMCs (Lonza)**			
**CC-2581 (Material) 0000658401 (Batch)**	52	Female	36 h

Supplementary Table 3. Demographics of blood donors, heritable PAH patients and healthy volunteers. Data represented as median (range).

		Control (n=5)	HPAH (n=5)
Females		4/5	3/5
Age (years)		29 (23- 56)	43 (30-58)
Time from diagnosis (months)		-	84 (29-171)
mPAP (mmHg)		-	58 (58-70)
Six minute walk distance (m)		-	366 (261-441)
WHO Functional Class	I	-	3
	II	-	1
Anticoagulants		-	3
Statins		-	1
ER Antagonists		-	5
PDE5 Inhibitors		-	5

Prostanoids	-	2

mPAP, mean Pulmonary Arterial Pressure; ER, Endothelin-Receptor; PDE5, Phosphodiesterase type 5. Treatment: at sampling.

Supplementary Table 4. PAH ECFC *BMPR2* genotype

Diagnosis	*BMPR2* Variant type	Variant
1.1: Idiopathic PAH	Stop gained	2:203383766 C>G
1.2.1: Heritable PAH - BMPR2	Missense variant	2:203329555 T>C
1.1: Idiopathic PAH	Splice region variant	NM_001204.6:c.418+5G>A
1.1: Idiopathic PAH	Frameshift	NM_001204.6c.1958_1 959delCT
1.1: Idiopathic PAH	Stop gained	NM_001204.6:c.2617C>T

Supplementary Table 5. RT reaction mix. Negative controls using the No-RT Control Mix (5x) we added into each plate to check for DNA/RNA contaminations. cDNA synthesis was completed using a SimpliAmp™ Thermal Cycler (Applied Biosystems, UK) with the following conditions:

Component	20 µL Reaction	Final Concentration
LunaScript RT SuperMix (5x)	4 µL	1x
RNA sample	variable	50 ng or 100 ng
Nuclease-free water	Remainder up to 20 µL	-

Supplementary Table 6. RT cycle.

Cycle Step	Temperature	Time	Cycles
Primer Annealing	25°C	2 minutes	1
cDNA Synthesis	55°C	10 minutes	1
Heat Inactivation	95°C	1 minute	1

Supplementary Table 7. qPCR master mix components and qPCR cycle

Component	10 µL Reaction	Final Concentration
Luna Universal qPCR Master Mix	5 **µL**	1X
10 µM forward primer	0.5 **µL**	0.25 µM
10 µM reverse primer	0.5 **µL**	0.25 µM
Nuclease-free water	3 **µL**	-

9 µL of the qPCR master mix and 1 µL of 1 ng/µL cDNA was added to each well of a 384-well skirted PCR plate (StarLab, UK, E1042-3840). A negative control for each gene of interest was added to the plate which contained 1 µL Nuclease-free water instead of 1 µL cDNA, plus 9 µL of appropriate qPCR master mix. Plates were sealed using a MicroAmp Optical Adhesive Film (Thermo Fisher Scientific, 4311971), inverted to mix samples and centrifuged for 1 minute at 1000 *g*. qPCR was performed using a QuantStudio 12K Flex Real-Time PCR System (Applied Biosystems, USA). Amplification was achieved over 40 cycles that consisted of the following conditions:

Cycle Stage	Temperature	Time	Cycles
Hold Stage	50°C	2 minutes	1
	95°C	5 minutes	
PCR Stage	95°C	15 seconds	40
	62°C	30 seconds	
Melt Curve Stage	95°C	15 seconds	1
	60°C	1 minute	
	95°C	15 seconds	

Supplementary Table 8. **List of primers**

**VEGFA**	Forward	AGGGAAAGGGGCAAAAACGAAAG
	Reverse	ACAAATGCTTTCTCCGCTCTG
**KLF2**	Forward	GTGAGAAGCCCTACCACTGCAACT
	Reverse	CCGGTTCTCTGGGTCCAATAAATA
**PECAM / CD31**	Forward	TGGAAGGAGTGCCCAGTCCCA
	Reverse	CGGAAGGATAAAACGCGGTCCTG
**BMPR2**	Forward	TCAAGAACGGCTATGTGCGTTT
	Reverse	TGTCCCCTTTTGATTTCTCCCAA
**KCNK1**	Forward	GGTGGTGGCCATCGTCCAT
	Reverse	GGCAATAAGGCCAAGTAGCAGG
**LDHA**	Forward	TGTAAAATACAGCCCGAACTGCAAG
	Reverse	TGGAATCTCCATGTTCCCCAAGG
**eNOS**	Forward	GCCGGAACAGCACAAGAGT
	Reverse	GAGGATGCCAAGGCCGC
**GAPDH**	Forward	CGGATTTGGTCGTATTGGGCG
	Reverse	GCCTTCTCCATGGTGGTGAAGAC

Supplementary Table 9. SOX17 binding sites in promoter regions of the investigated genes.

Gene name	GeneHancer Identifier	Genomic Location	Predicted SOX17 binding sites
Prostaglandin I2 Synthase (*PTGIS*)	GH20J049565	chr20:49565626-49571680	8
Prostaglandin Reductase 2 (*PTGR2*)	GH14J073849	chr14:73849846-73853324	5
Superoxide dismutase 1 (*SOD1*)	GH21J031658	chr21:31658378-31661263	0
Dickkopf WNT Signaling Pathway Inhibitor 2 (*DKK2*)	GH04J107033	chr4:107032470-107038386	9
Dickkopf WNT Signaling Pathway Inhibitor 2 (*DKK2*)	GH04J107283	chr4:107282283-107288570	8

Supplementary Table 10. SOX17 binding sites in putative enhancer regions of the investigated genes.

Region name	Genomic location	Predicted SOX17 binding sites
PTGIS enhancer 1	chr20:49560623-49564067	6
PTGIS enhancer 2	chr20:49593864-49597044	5
SOD1 enhancer 1	chr21:31629650-31630816	0
SOD1 enhancer 2	chr21:31644308-31645388	1
DKK2 enhancer 1	chr4:107058473-107061289	5

DKK2 enhancer 2	chr4:107067359-107072255	8
DKK2 enhancer 3	chr4:107117489-107123773	13
DKK2 enhancer 4	chr4:107126634-107132005	9
DKK2 enhancer 5	chr4:107277886-107280956	10

Supplementary Table 11. Primary and secondary antibodies used for immunostaining.

Name	Species	Company	Cat No	Dilution
AlexaFluor488 (Anti-Rabbit)	Goat	Life Technologies	A11034	1:300
VE-Cadherin (AlexaFluor 488 Conjugated)	Mouse	eBioscience	53-1449-42	1:100
α-Smooth Muscle Actin (AlexaFluor 647 Conjugated)	Mouse	Abcam	ab196919	1:100
Smooth muscle calponin	Rabbit	Abcam	ab46794	1:100
Alexa fluor-546 (Anti-Rabbit)	Goat	Life Technologies	A11010	1:100
Alexa Fluor Plus 594 (Anti-Mouse)	Goat	Life Technologies	A32742	1:300
Alexa Fluor Plus 594 (Anti-Rabbit)	Goat	Life Technologies	A32740	1:300
ERG (Anti-human)	Rabbit	Abcam	ab92513	1:100
vWF (Anti-human)	Rabbit	Dako	A0082	1:500
α-Smooth Muscle Actin (Anti-human)	Mouse	Dako	M0851	1:1000
Anti-KCNK1 (anit-human)	Rabbit	Life Technologies	PA541010	1:50
FITC Anti-Rabbit	Goat	Jackson ImmunoResearch Inc	111-095-003	1:100
TRITC Anti-Mouse	Goat	Sigma-Aldrich	T5393	1:100

Supplementary Supplementary Table 12. Antibodies Used For Western blotting.

Name	Species	Company	Cat No	Dilution
human β-actin	Mouse	Santa Cruz	sc-47778	1:2000
human BMPR2	Mouse	BD Biosciences	612292	1:1000
HRP-labelled anti-mouse	Sheep	GE Healthcare	NA931	1:2000

SUPPLEMENTARY FIGURES (RESULTS)

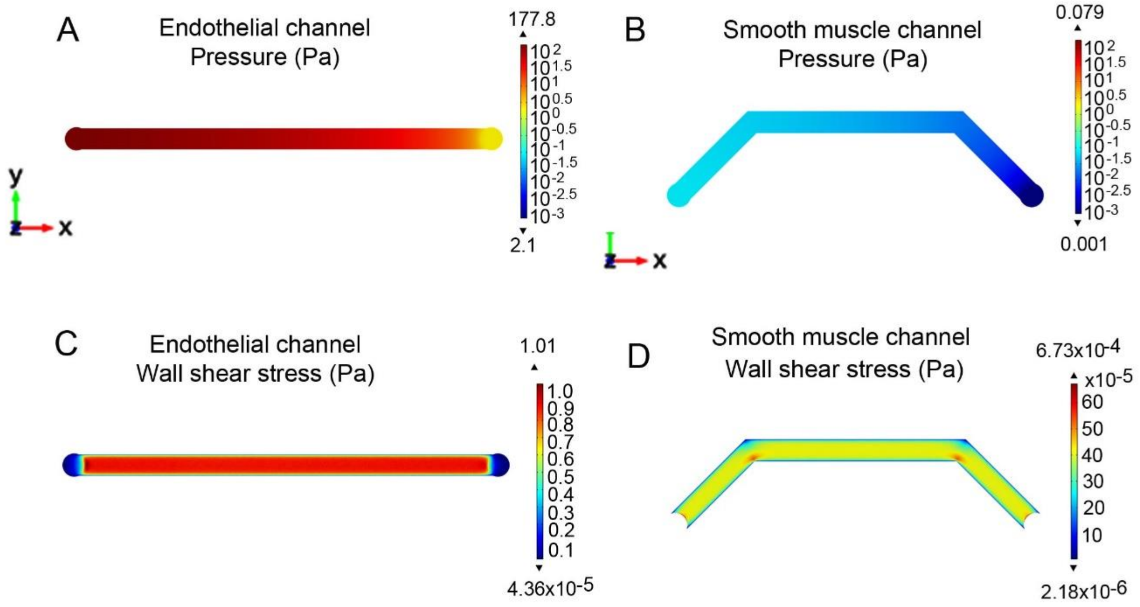

Supplementary Figure 1. Pressure changes in microfluidic channels of PA-on-a-chip in a rigid PDMS wall model. A) Pressure field acting on ECs. B) Pressure field acting on SMCs. C) WSS acting on ECs and D) WSS acting on SMCs. Volumetric flow rate in endothelial channel QEC = 0.35 mL/min and in smooth muscle channel QSMC=10 µL/h, with outlet tube diameter and length of 1 mm and 1.6 mm respectively. Changes in pressure field and wall shear stress as presented as 2D colour maps, COMSOL modelling.

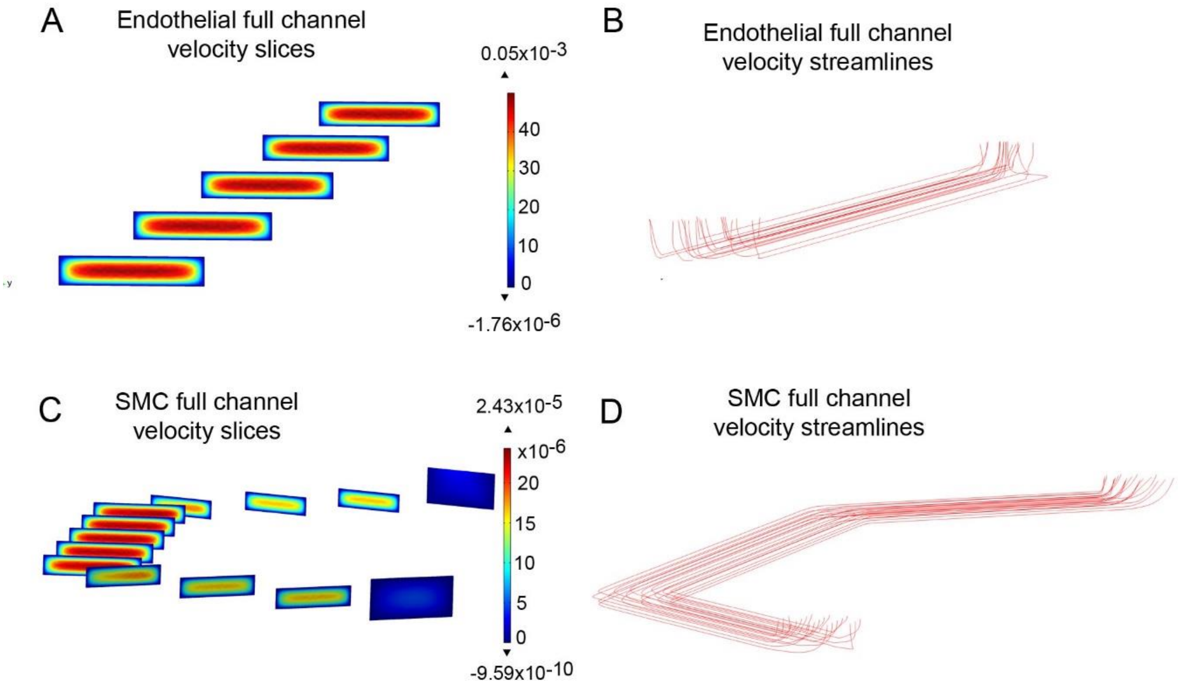

Supplementary Figure 2. Visual representations of the velocity field in microfluidic channels of PA-on-a-chip. A) Velocity slices perpendicular to flow direction in the endothelial channel. B) Velocity streamlines of laminar flow within endothelial channel. C) Velocity slices perpendicular to flow direction in the SMC channel. D) Velocity streamlines of laminar flow within the SMC channel. Changes in velocity field in (A and C) are presented as 2D colour maps, with units ms-1 of fluid through the channel, assuming that volumetric flow rate in endothelial and smooth muscle channel is QEC = 0.35 mL/min, and QSMC = 10 µL/h, respectively; COMSOL modelling.

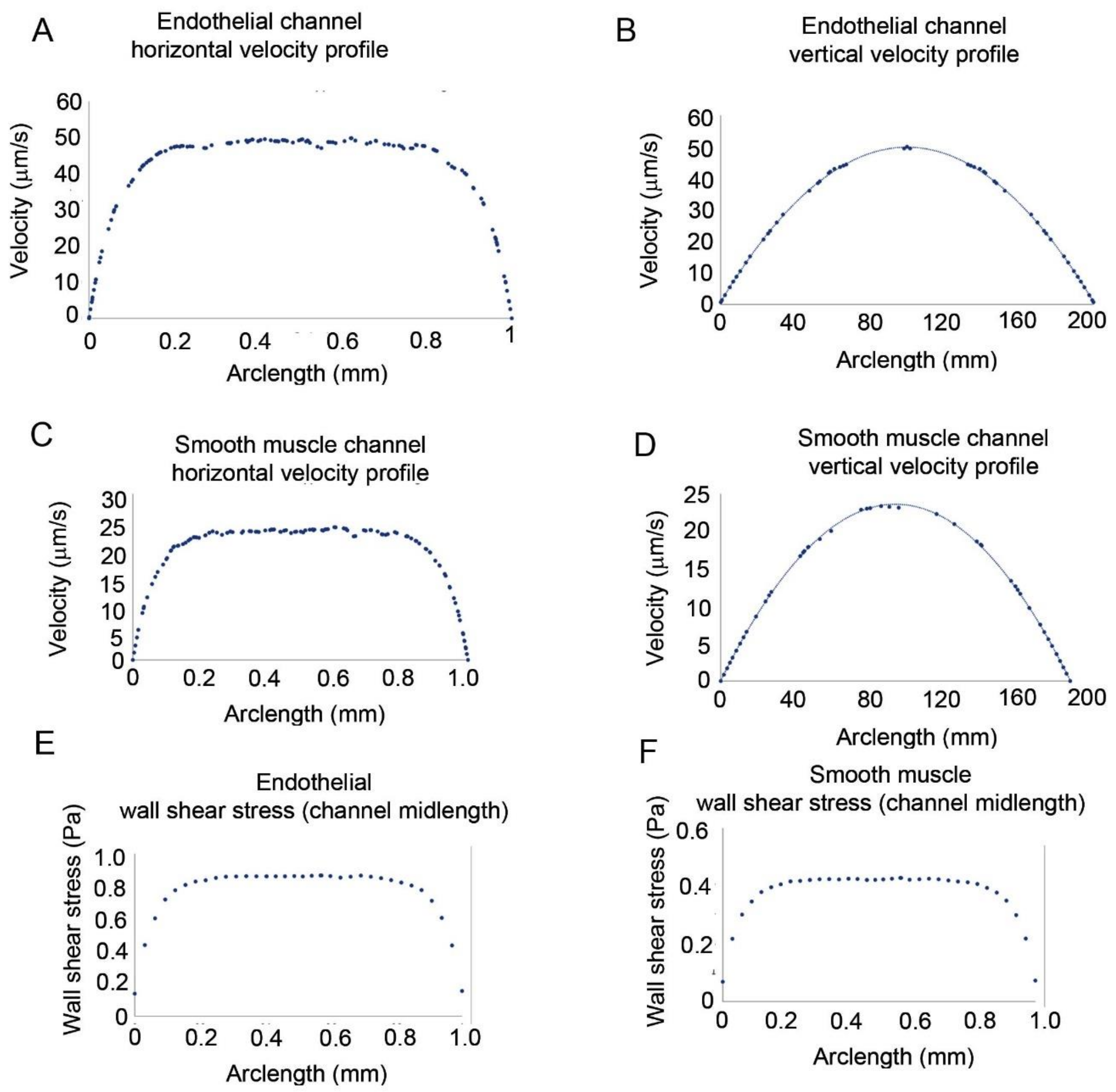

Supplementary Figure 3. Velocity profiles along vertical and horizontal lines of symmetry in the channel cross section in PA-on-a-chip. A) EC channel horizontal velocity profile. B) EC channel vertical velocity profile. C) SMC channel horizontal velocity profile. D) SMC channel vertical velocity profile. E) EC channel floor WSS acting on ECs at channel mid-length. F) SMC channel ceiling WSS acting on SMCs at channel mid-length. The analysis was done in COMSOL, at the mid-channel cross section, with volumetric flow rate QEC = 0.35 ml/min and QSMC=10 µl/h.

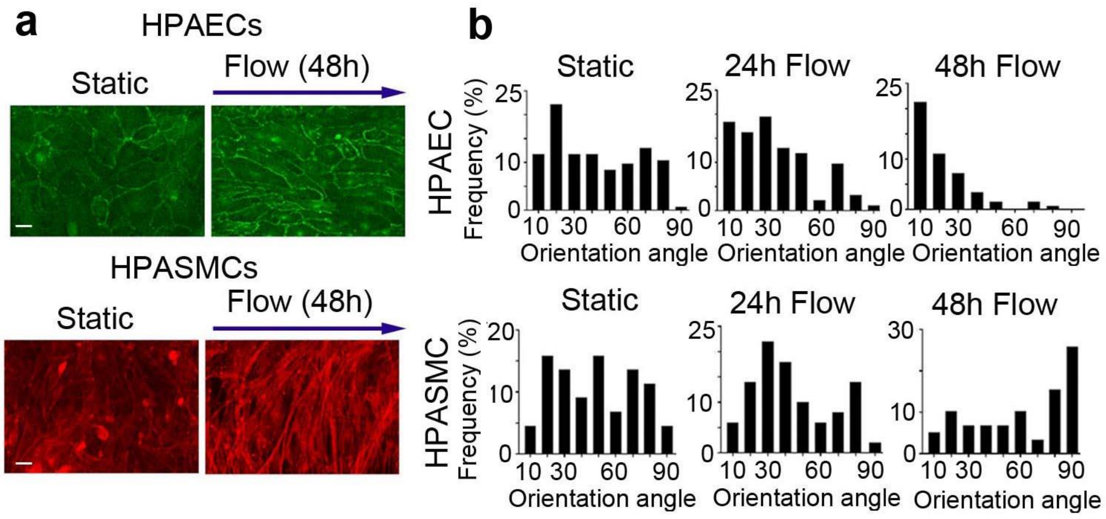

Supplementary Figure 4. Morphology of HPAECs and HPASMCs under flow, 48h in culture. (**a**) Confocal images of HPAECs and HPASMC. HPAECs were cultured under flow for 48 hours, the arrow shows the direction of flow. VE-cadherin: green, α-smooth muscle actin: red. Bar=10 µm. (**b**) Alignment (mean orientation angle to the flow direction) of endothelial and smooth muscle cells at different time points of flow exposure, as indicated. Cell orientation was measured in 3-4 different areas within a chip in 3 chips/group (~300 cells/chip).

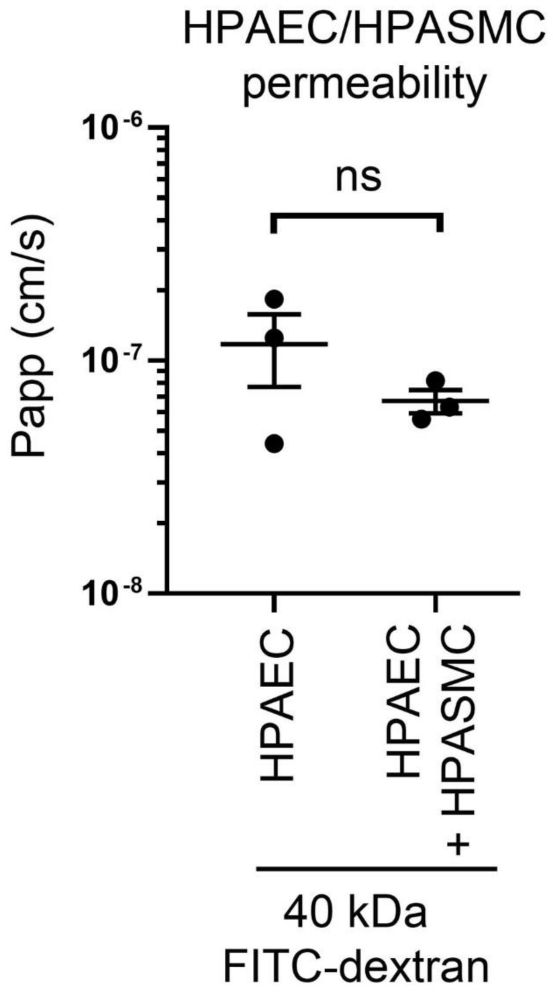

Supplementary Figure 5. Contributions of HPASMCs to changes in endothelial barrier function in PA-on-a-chip. Passage of 40 kDa (1 mg/mL; 1h) across a monolayer of HPAECs cultured with, or without HPASMCs, was used as a measure of barrier function in PA-on-a-chip. (n=3). Error bars indicate mean ±SEM of a one-way ANOVA with a Tukey's post-hoc correction test.

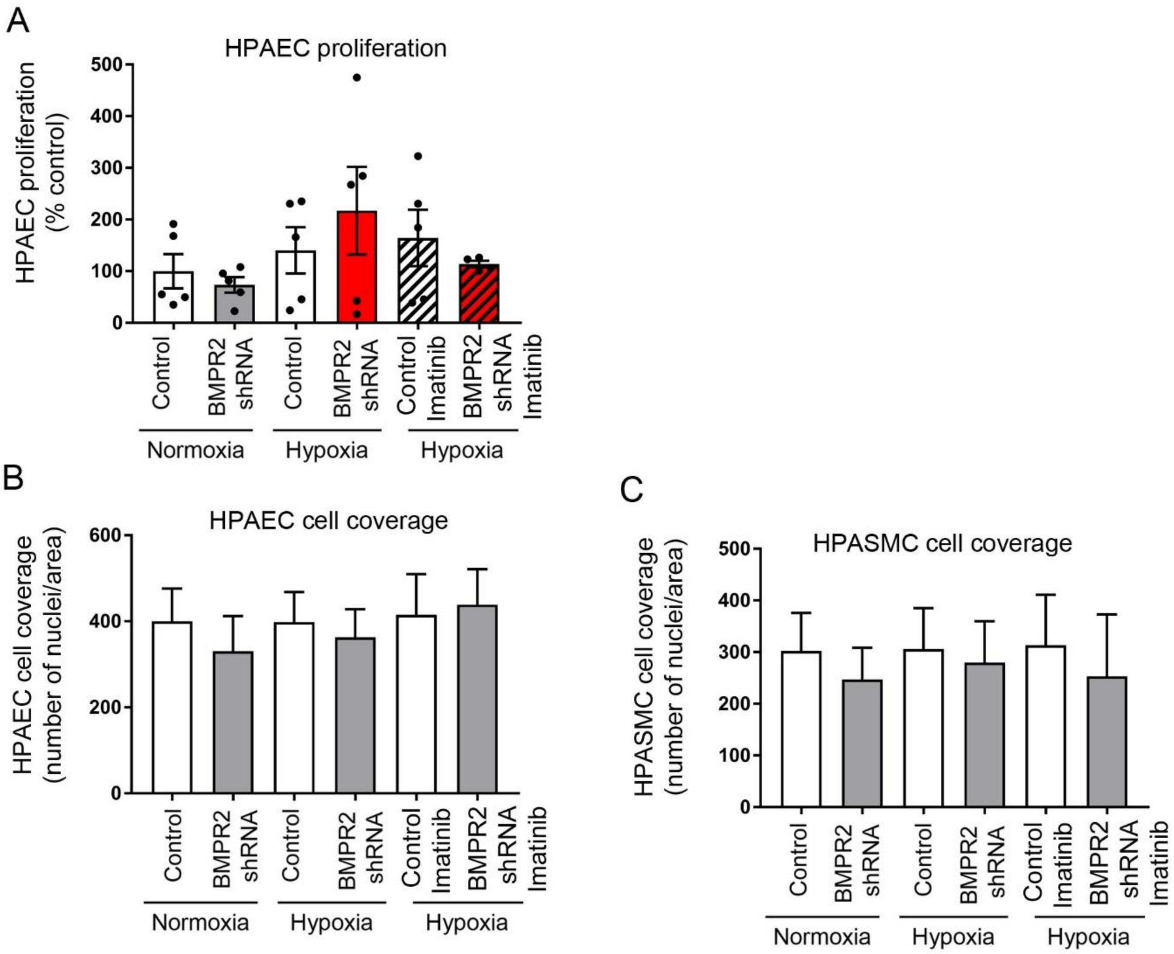

Supplementary Figure 6. Endothelial proliferation and cell coverage. (**A**) Effect of hypoxia and endothelial BMPR2 knockdown on HPAEC proliferation. Cells were untreated or were treated with 10 µM imatinib mesylate for 24h and cell proliferation was measured in EdU assay. (**B**) HPAEC coverage per area (0.5 mm^2). Cell coverage was defined as a number of cell nuclei/area. (**C**) HPASMC coverage per area. n=4-5 individual chips.

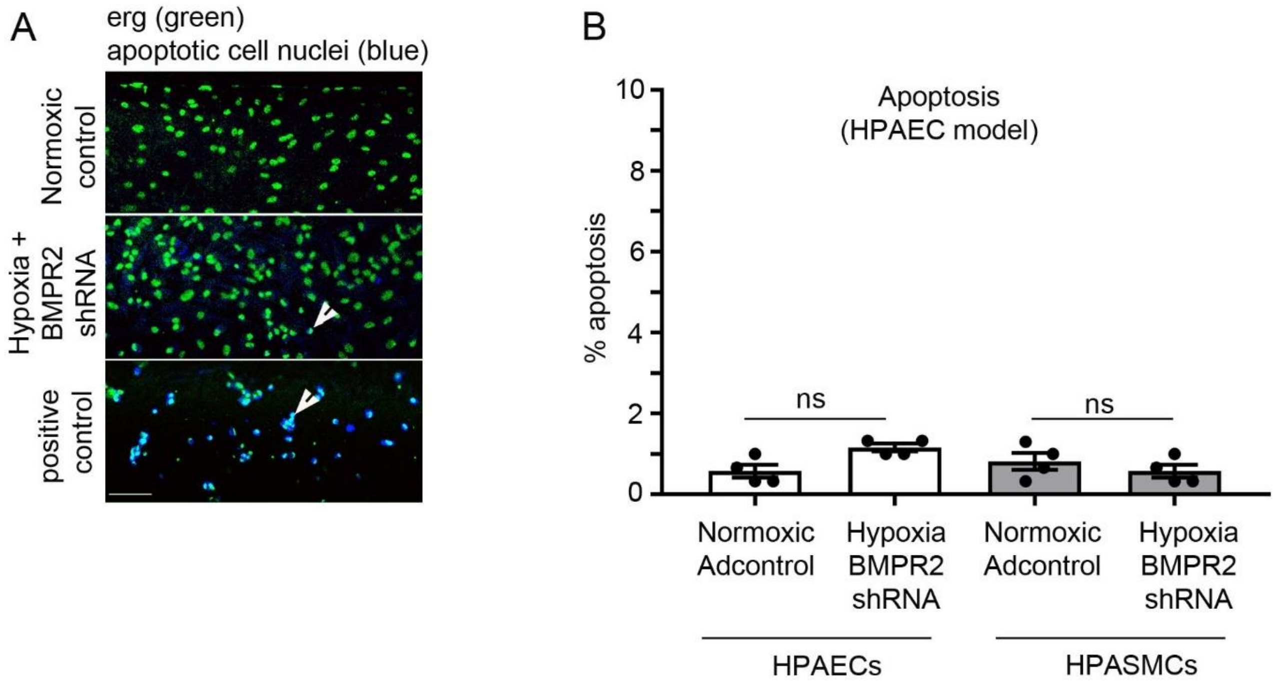

Supplementary Figure 7. Effect of hypoxia and BMPR2 knockdown on endothelial and smooth muscle cell apoptosis in the HPAEC "double hit" model. (**A**) Representative confocal images of HPAEC channel, with apoptotic cell nuclei labelled blue (Click-iT Plus TUNEL Alexa Fluor 647) and endothelial cell nuclei labelled green (immunofluorescence, anti-erg antibody) in cells treated, as indicated. Cells treated with DNAse I were used as positive control. Arrowheads point to apoptotic endothelial cells. Bar=100 µm. (**B**) Graph showing % of apoptotic cells in HPAEC and HPASMC channel. Ns-non-significant, unpaired Student t-test, n=4. Apoptotic HPAECs were identified by blue erg-positive nuclei, while apoptotic HPASMCs were marked by blue nuclei that were erg-negative. All cell nuclei (HPASMCs and HPAECs) were labelled with Hoechst (not shown).

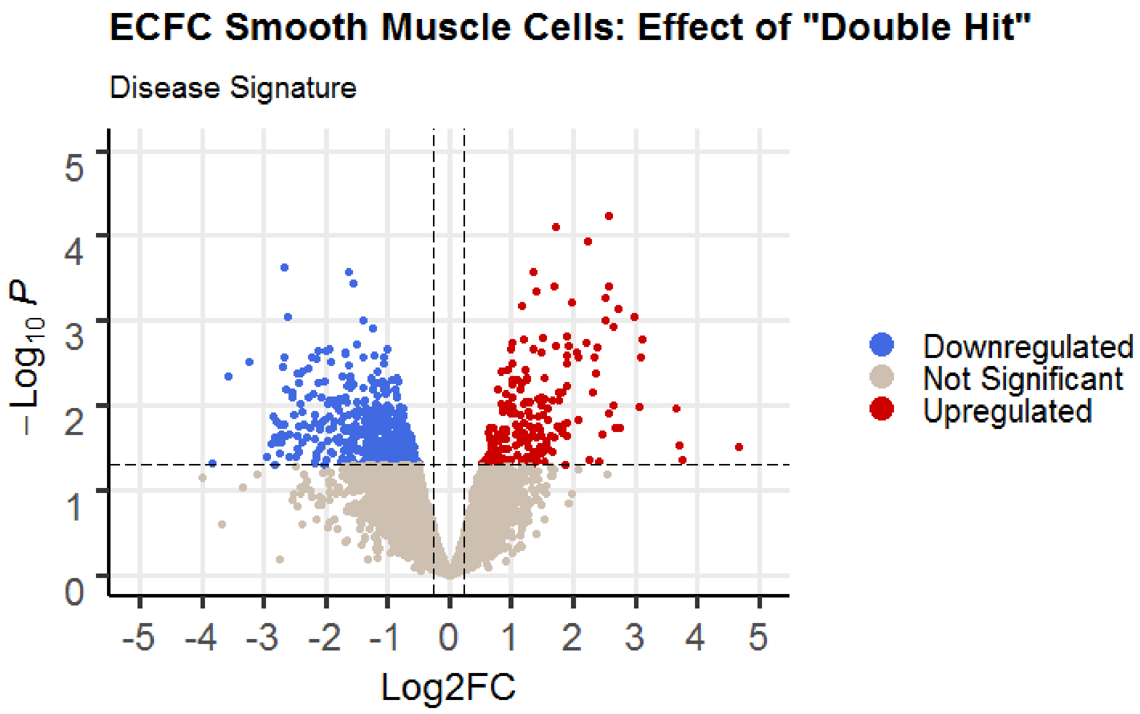

Supplementary Figure 8. Differentially expressed genes in WT HPASMCs co-cultured with BMPR2-deficient HPAECs. Volcano plot shows differentially expressed genes, with red dots marking upregulated genes and blue dots marking downregulated genes, as indicated. n=5 different biological donors/treatment group.

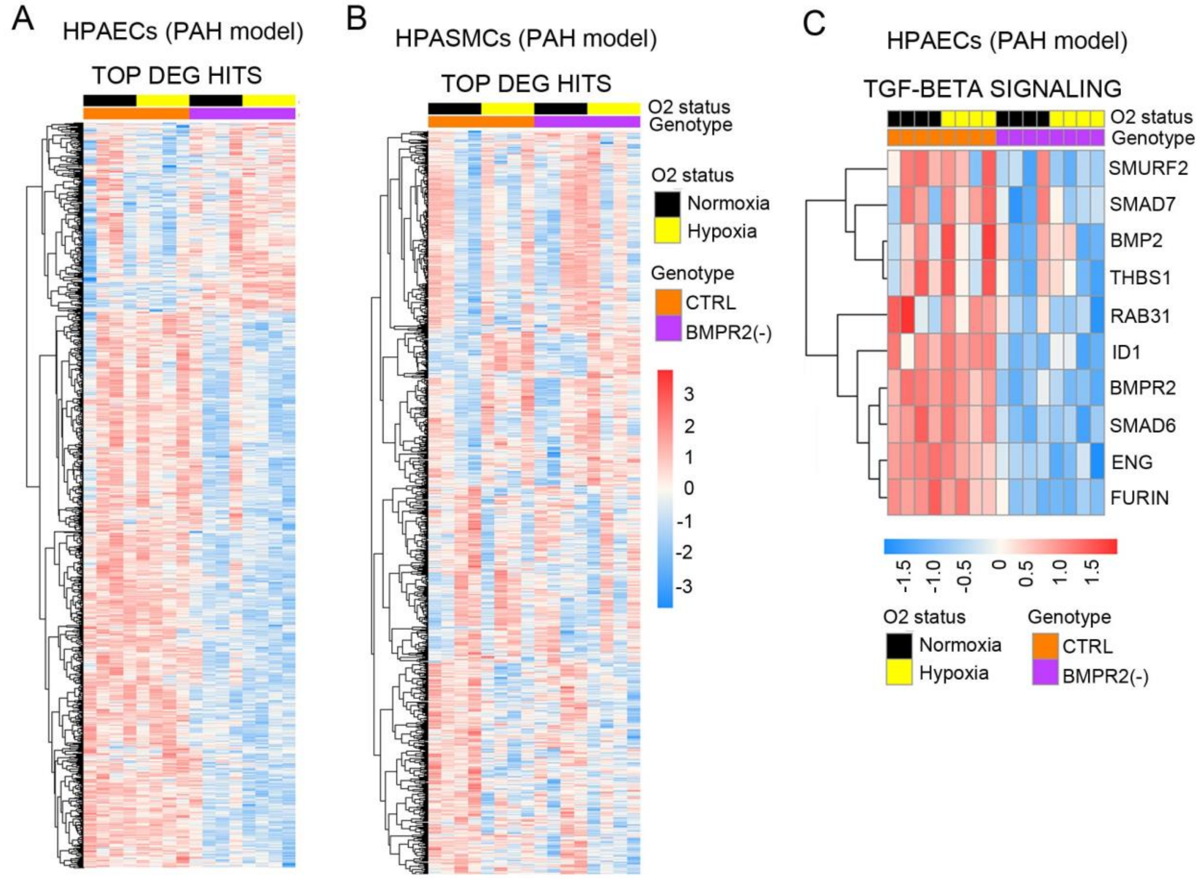

Supplementary Figure 9. Disease gene signatures in the "double hit" microfluidic model of PAH. (A, B) Heatmaps with hierarchical DEG clustering in (A) HPAECs and in (B) HPASMCs from the "double hit" microfluidic PAH model. (C) Heatmap of genes involved in the TGF-β signalling pathway in HPAECs. The genotype status (control and BMPR2 knockdown) and oxygen status (normoxia or hypoxia) shown at the top of the heatmap are colour-coded, as indicated. Changes in gene expression are also colour coded, with blue denoting a lower relative gene expression and red denoting a higher relative gene expression. Each column represents 1 experimental repeat (n=4/treatment group).

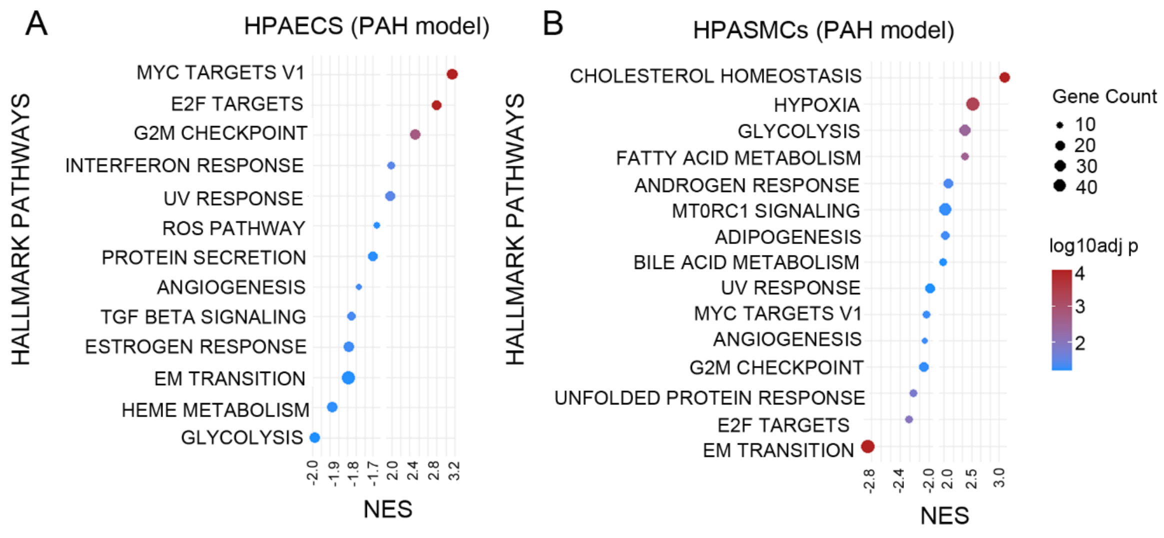

Supplementary Figure 10. Gene enrichment scores in GSEA pathway analysis in the "double hit" microfluidic model of PAH. (**A**) Dot plot matrix of GSEA pathway analysis in HPAECs; (**B**) Dot plot matrix of GSEA pathway analysis in HPASMCs.

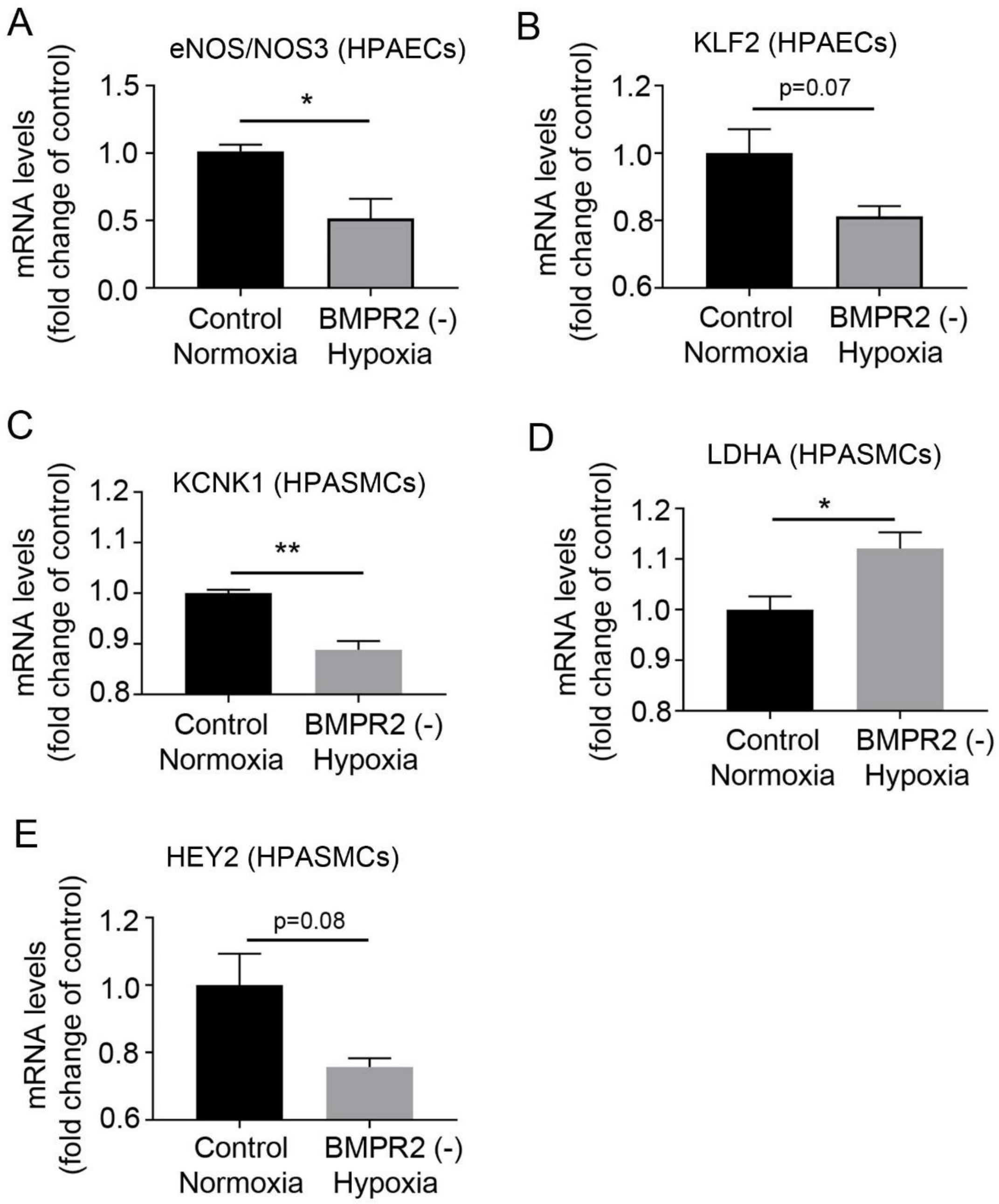

Supplementary Figure 11. qPCR validation of selected DE genes identified in HPAECs and HPASMCs in the "double hit" microfluidic PAH model. mRNA expression of selected endothelial (**A, B**) and smooth muscle (**C, D, E**) differentially expressed genes assessed by qPCR. **P<0.01; *P<0.05; Unpaired t test. n=3.

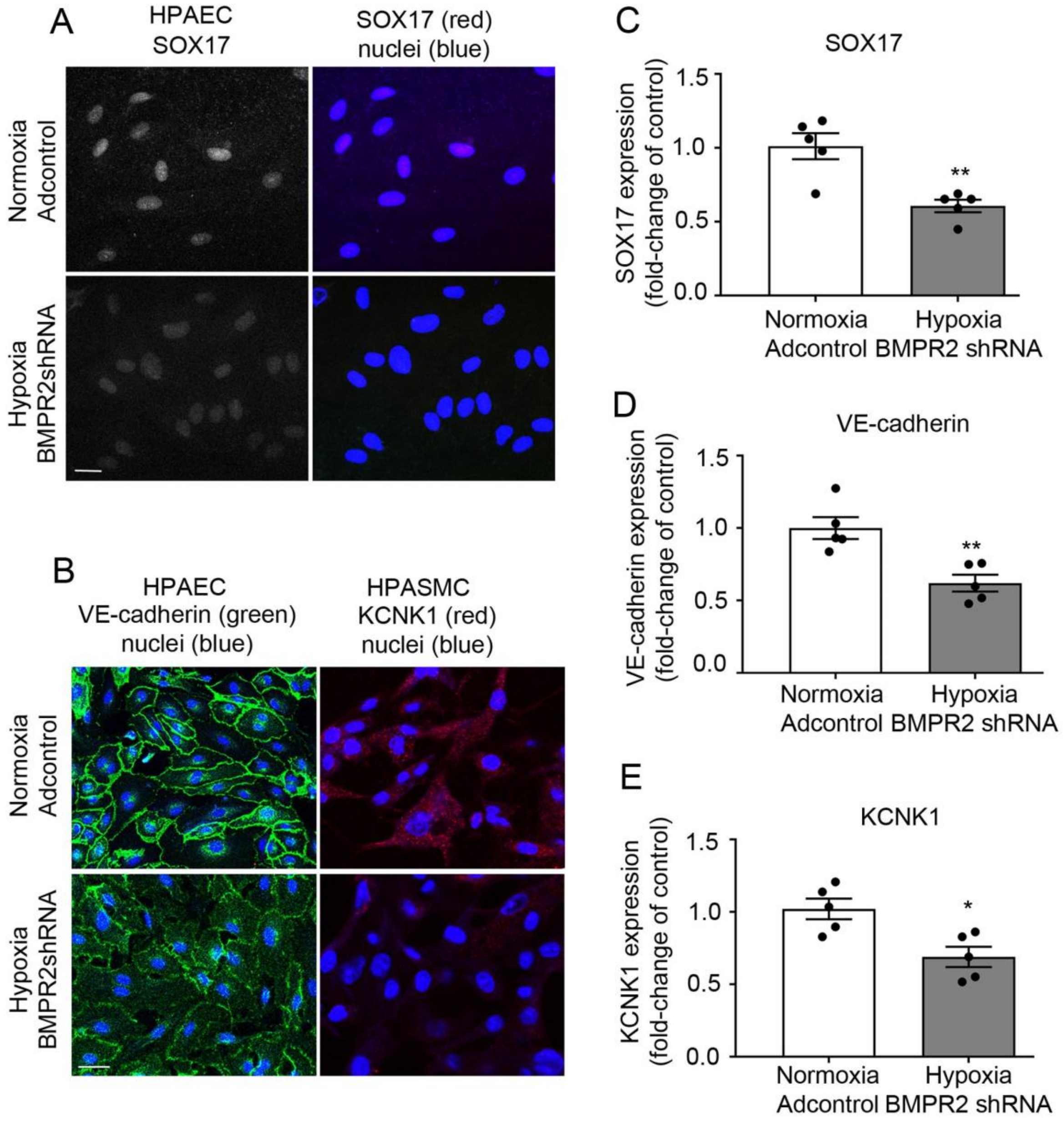

Supplementary Figure 12. Expression changes of selected protein targets in HPAECs and HPASMCs in the "double hit" microfluidic PAH model. (**A**) and (**B**) are representative confocal images of fluorescently labelled SOX17, VE-cadherin in HPAECs and KCNK1 in HPASMCs, treated, as indicated. Bar=10 µm. (**C, D, E**) are corresponding graphs showing changes in protein expression of these targets in normoxic controls (Normoxia Adcontrol) and cells under the "double hit" conditions (Hypoxia BMPR2 shRNA). Semi-quantitative analysis of fluorescence intensity. Error bars indicate mean ±SEM, unpaired t-test, n=5.

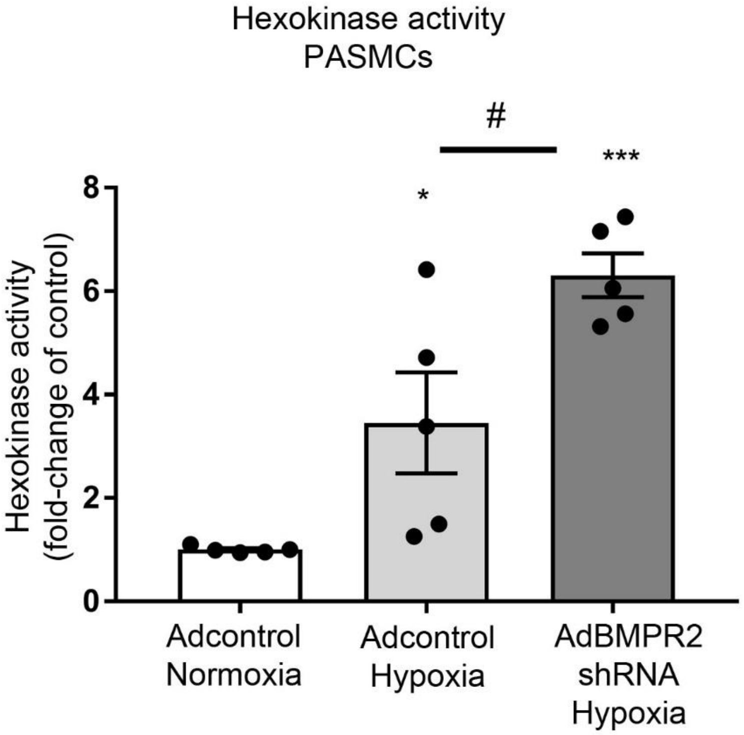

Supplementary Figure 13. Hexokinase activity in PASMCs under noromoxic, hypoxic and "double hit" conditions. Hexokinase activity was measured in pulled samples of PASMCs (1.5 x10^5 cells/treatment) co-cultured with HPAECs under normoxic conditions (Adcontrol Normoxia), under hypoxia (Adcontrol Hypoxia) and under the "double hit" conditions (AdBMPR2 shRNA Hypoxia). Hexokinase activity under basal, control conditions was 0.86 ± 0.043 nmol/min/mL. Error bars indicate mean ±SEM of a one-way ANOVA with a Tukey's post-hoc correction test, n=5.

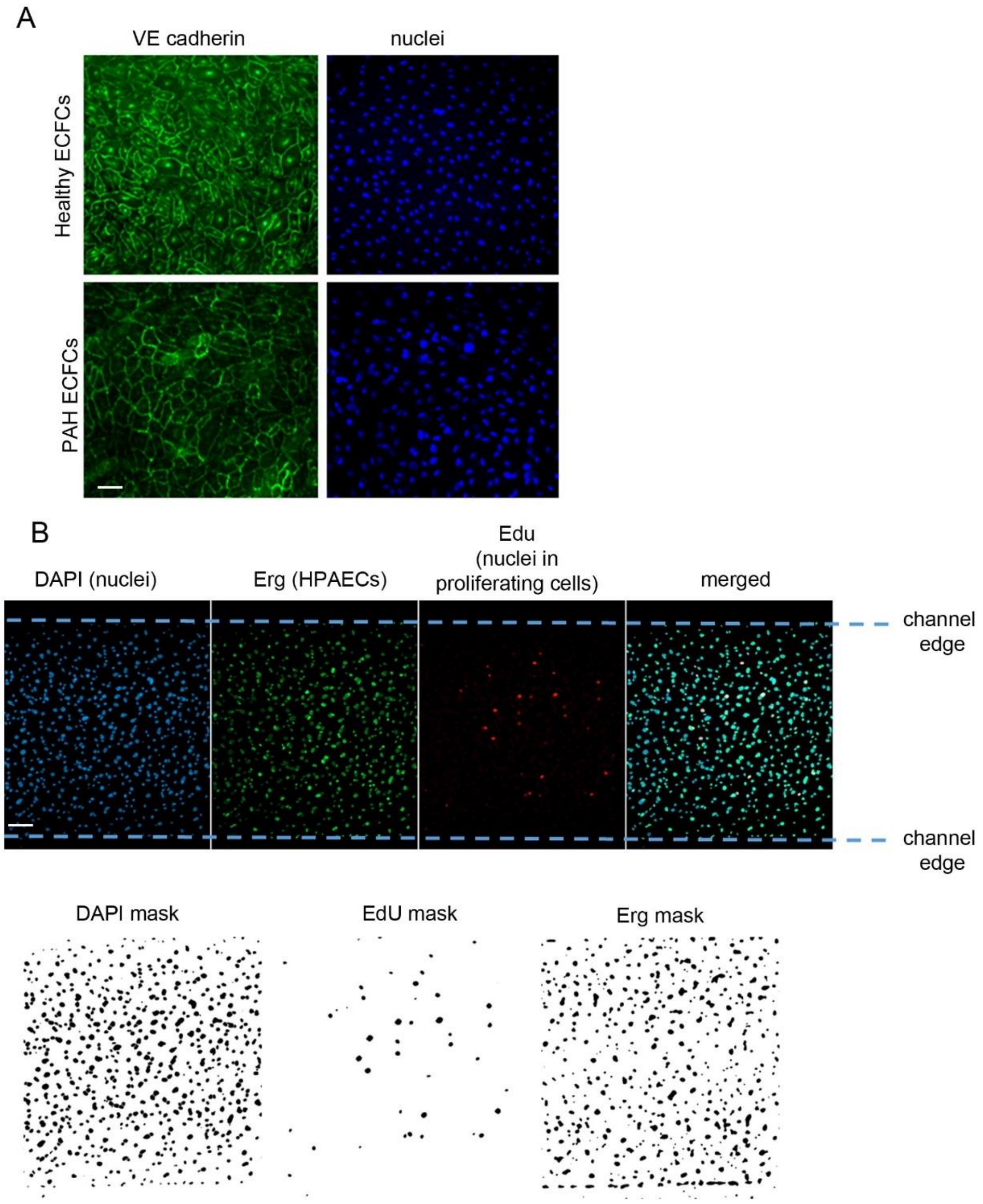

Supplementary Figure 14. ECFC characteristics and cell counting method. (**A**) VE Cadherin immunostaining in ECFCs from healthy and PAH individuals. VE-cadherin is green and cell nuclei are blue, as indicated. Bar=50µm. (**B**) Representative images of images used for cell counting. Endothelial cell nuclei are green (Erg-positive) and all cell nuclei are blue (DAPI). Colour channels were converted to binary masks and overlaid onto a DAPI mask for semi-automated counting. Bar=100 µm.

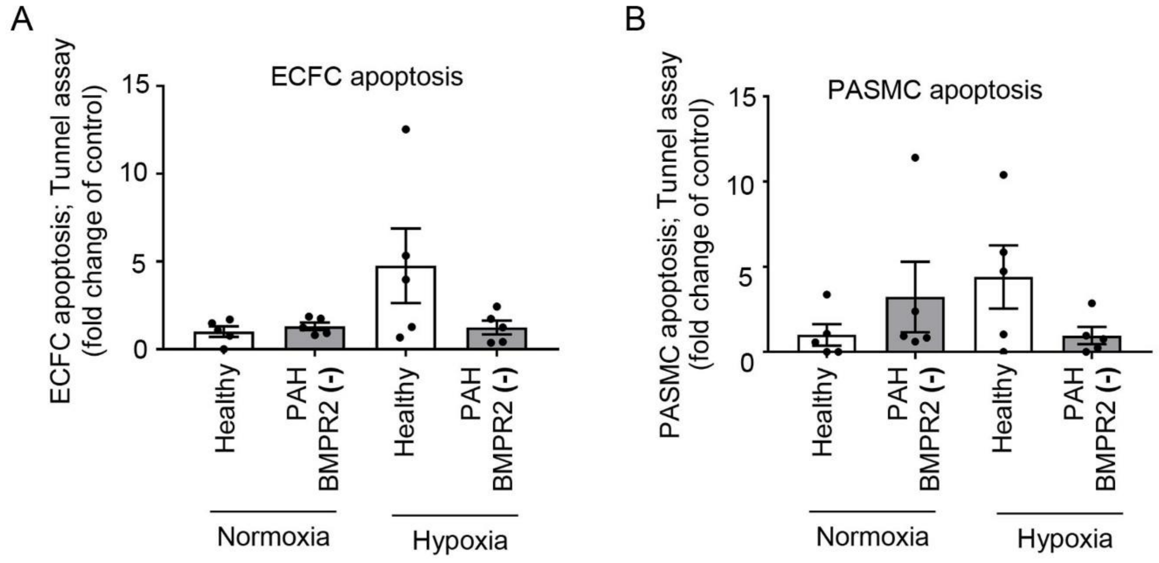

Supplementary Figure 15. Cell apoptosis in the ECFC PAH model. (**A**) Apoptosis of healthy and PAH ECFCs in PA-on-a-chip under normoxic or hypoxic conditions (2% O_2, 24hrs), as indicated. (**B**) Apoptosis of HPASMCs co-cultured with control or patient ECFCs under normoxic or hypoxic conditions. n=4-5 biological donors, each assayed in a separate chip. Bars are means ±SEM; one-way ANOVA with a Tukey post-test.

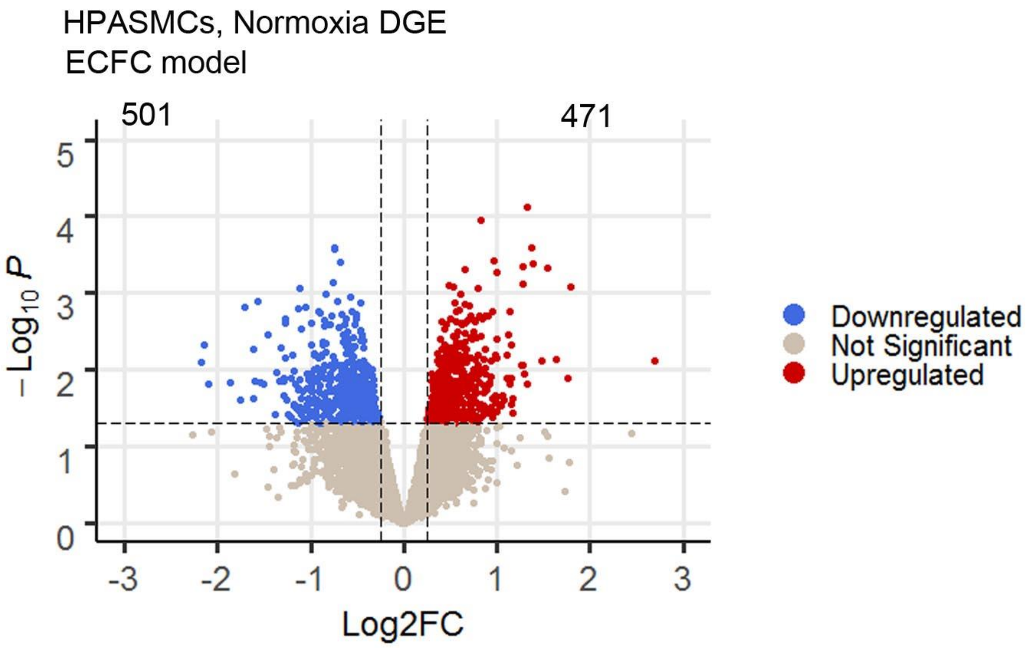

Supplementary Figure 16. Differentially expressed genes in WT HPASMCs co-cultured with PAH ECFCs. Volcano plot showing differentially expressed genes. n=5 different biological donors/treatment group.

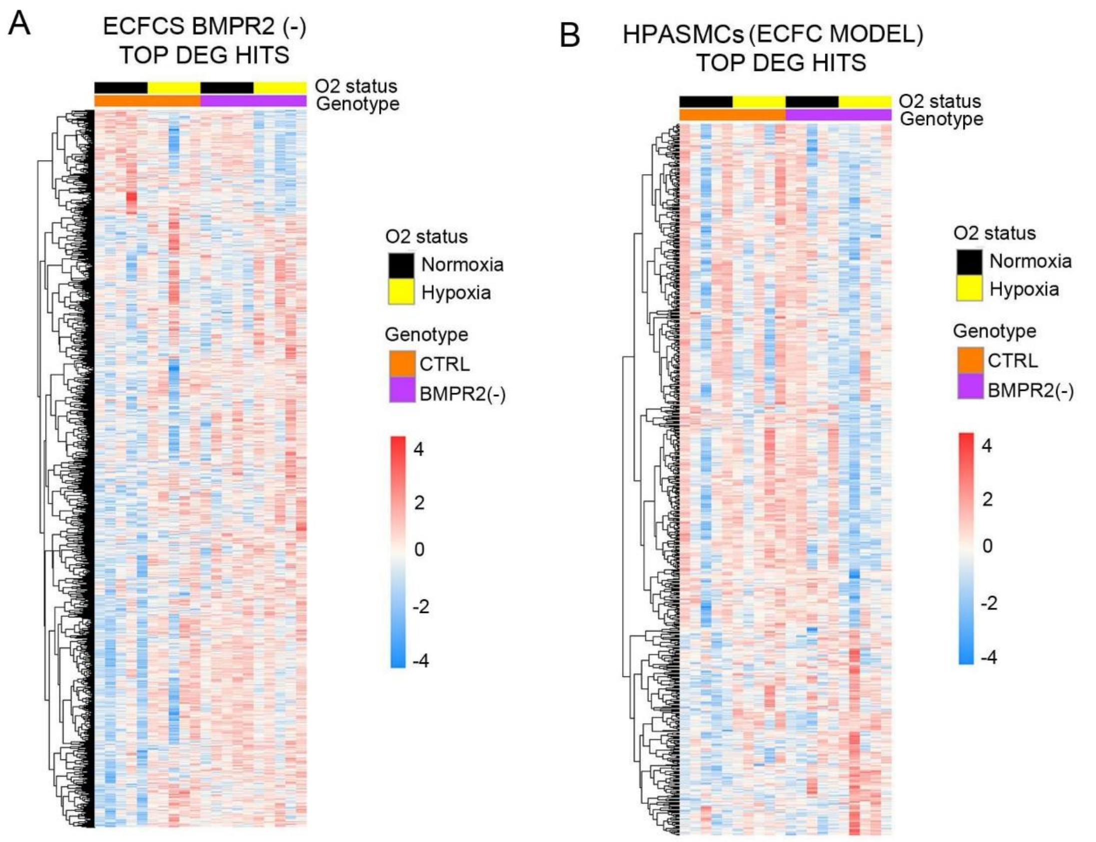

Supplementary Figure 17. Disease gene signatures in the ECFC microfluidic model of PAH. Heatmaps with hierarchical gene clustering in (**A**) healthy and PAH ECFCs (**B**) HPASMC co-cultured with these cells. The genotype status (control and BMPR2 knockdown) and oxygen status (normoxia or hypoxia) are shown at the top of each heatmap in different colours, as indicated. Changes in gene expression are also colour coded, with blue denoting a lower relative gene expression and red denoting a higher relative gene expression. Each column represents 1 donor; n=5 different biological donors/treatment group.

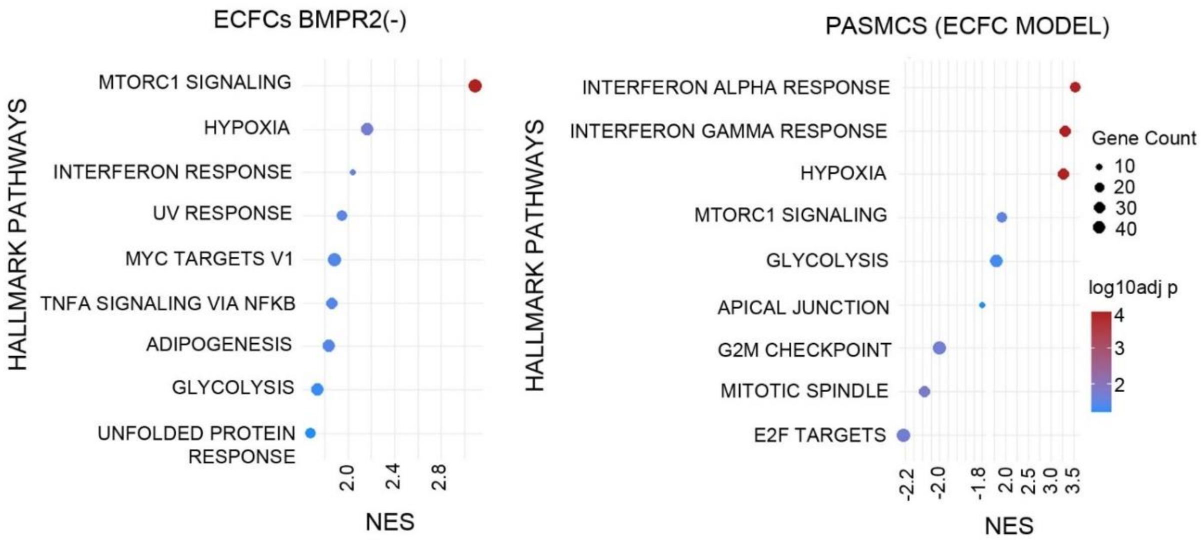

Supplementary Figure 18. Dot plot matrix of GSEA pathway analysis. (**A**) Dot plot matrix of GSEA pathway analysis generated from healthy or PAH ECFCs cultured under normoxic or hypoxic conditions. (**B**) Dot plot matrix of GSEA pathway analysis generated from HPASMCs co-cultured with healthy or PAH ECFCs.

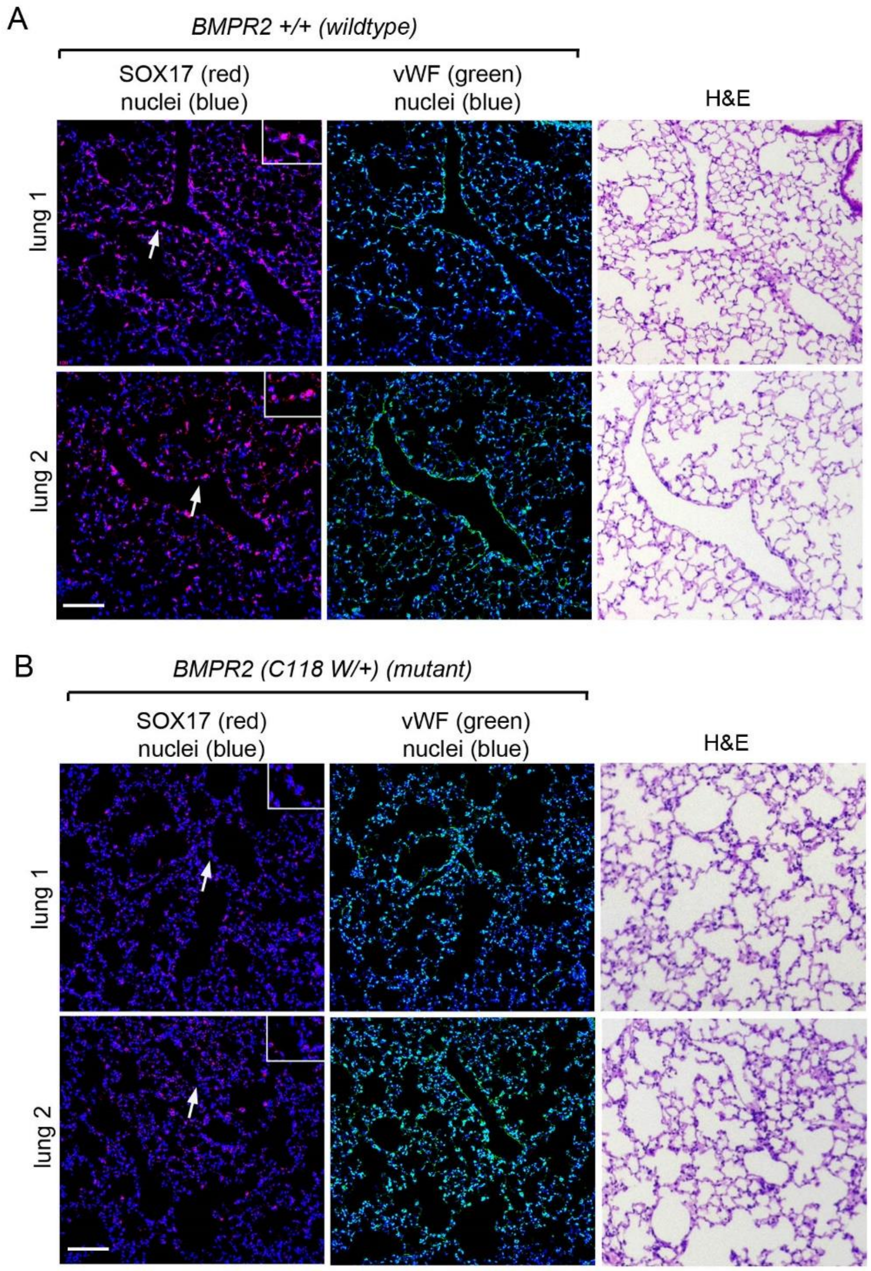

Supplementary Figure 19. Loss of endothelial SOX17 in BMPR2-deficient mice. (**A**) Lung tissues from wildtype mice and (**B**) Lung tissues from BMPR2-deficient heterozygous BMPR2$^{C118+/-}$ mice were stained for SOX17 (red), vWF (green) and nuclei (blue), as indicated. Haematoxylin and eosin (H&E) staining of corresponding sequential sections is shown on the right, as indicated. (**A, B**) are representative images of n=4/group. Bar=50 µm.

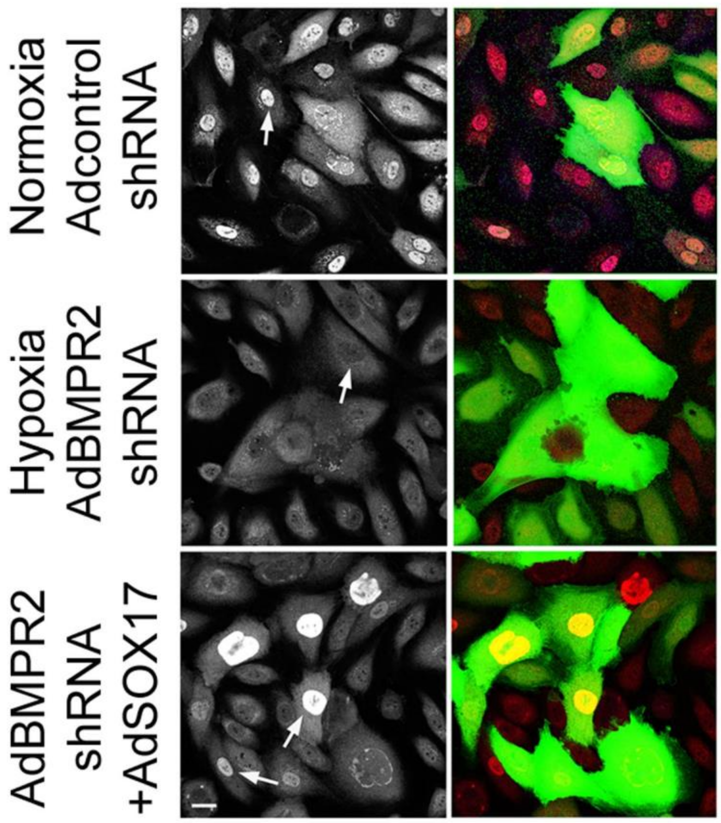

Supplementary Figure 20. Localization of SOX17 in HPAECs. Localization of SOX17 in BMPR2-deficient HPAECs (AdBMPR2 shRNA), controls (Adcontrol shRNA) and BMPR2-deficient cells overexpressing SOX17 (AdBMPR2 shRNA + AdSOX17), as indicated. Arrows point to cell nuclei. Bar=10 μm

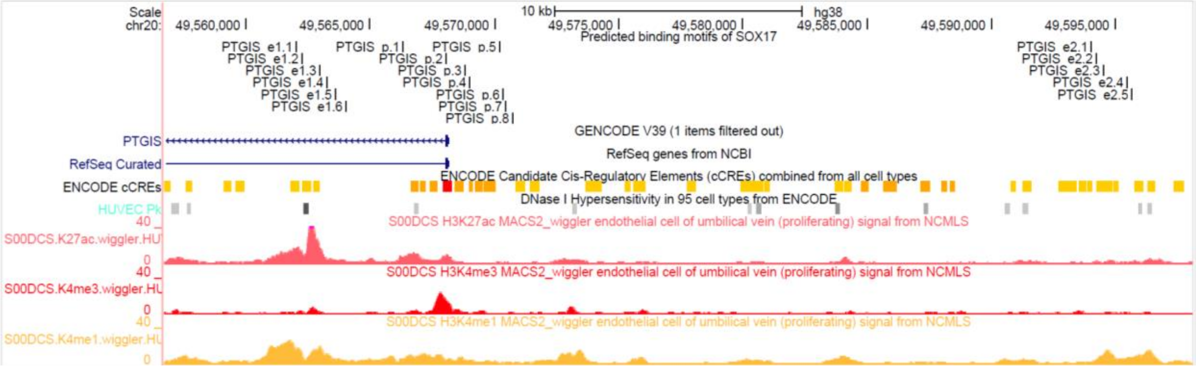

Supplementary Figure 21. Genetic locus of PTGIS. Integration of ENCODE cCREs track indicates candidate cis-regulatory elements (promoters in red, enhancers in yellow), DNase I sensitivity (indicates accessible chromatin) from hUVECs, H3K27Ac (indicates active enhancers), H3K4Me3 (indicates promoters) and H3K4Me1 (indicates enhancers) from hUVECs. All the predicted binding sites of SOX17 in the two putative enhancers and promoter regions of PTGIS are presented in the 2nd track (black bars e.g. PTGIS e1.1 indicates enhancer 1 binding site 1). Image from UCSC browser, assembly hg38.

SUPPLEMENTARY REFERENCES

1. Fishilevich, S., Nudel, R., Rappaport, N., Hadar, R., Plaschkes, I., Iny Stein, T., Rosen, N., Kohn, A., Twik, M., Safran, M.*, et al.* (2017). GeneHancer: genome-wide integration of enhancers and target genes in GeneCards. Database (Oxford) *2017*.
2. Stelzer, G., Rosen, N., Plaschkes, I., Zimmerman, S., Twik, M., Fishilevich, S., Stein, T. I., Nudel, R., Lieder, I., Mazor, Y.*, et al.* (2016). The GeneCards Suite: From Gene Data Mining to Disease Genome Sequence Analyses. Curr Protoc Bioinformatics *54*: 1 30 31-31 30 33.
3. Kent, W. J., Sugnet, C. W., Furey, T. S., Roskin, K. M., Pringle, T. H., Zahler, A. M., and Haussler, D. (2002). The human genome browser at UCSC. Genome Res *12*: 996-1006.
4. Balmer, P., Bauer, A., Pujar, S., McGarvey, K. M., Welle, M., Galichet, A., Muller, E. J., Pruitt, K. D., Leeb, T., and Jagannathan, V. (2017). A curated catalog of canine and equine keratin genes. PLoS One *12*: e0180359.
5. Consortium, E. P., Moore, J. E., Purcaro, M. J., Pratt, H. E., Epstein, C. B., Shoresh, N., Adrian, J., Kawli, T., Davis, C. A., Dobin, A.*, et al.* (2020). Expanded encyclopaedias of DNA elements in the human and mouse genomes. Nature *583*: 699-710.
6. Thurman, R. E., Rynes, E., Humbert, R., Vierstra, J., Maurano, M. T., Haugen, E., Sheffield, N. C., Stergachis, A. B., Wang, H., Vernot, B.*, et al.* (2012). The accessible chromatin landscape of the human genome. Nature *489*: 75-82.
7. Adams, D., Altucci, L., Antonarakis, S. E., Ballesteros, J., Beck, S., Bird, A., Bock, C., Boehm, B., Campo, E., Caricasole, A.*, et al.* (2012). BLUEPRINT to decode the epigenetic signature written in blood. Nat Biotechnol *30*: 224-226.

8. Weirauch, M. T., Yang, A., Albu, M., Cote, A. G., Montenegro-Montero, A., Drewe, P., Najafabadi, H. S., Lambert, S. A., Mann, I., Cook, K.*, et al.* (2014). Determination and inference of eukaryotic transcription factor sequence specificity. Cell *158*: 1431-1443.
9. Wojciak-Stothard, B., Abdul-Salam, V. B., Lao, K. H., Tsang, H., Irwin, D. C., Lisk, C., Loomis, Z., Stenmark, K. R., Edwards, J. C., Yuspa, S. H.*, et al.* (2014). Aberrant chloride intracellular channel 4 expression contributes to endothelial dysfunction in pulmonary arterial hypertension. Circulation *129*: 1770-1780.
10. Maoz, B. M., Herland, A., FitzGerald, E. A., Grevesse, T., Vidoudez, C., Pacheco, A. R., Sheehy, S. P., Park, T. E., Dauth, S., Mannix, R.*, et al.* (2018). A linked organ-on-chip model of the human neurovascular unit reveals the metabolic coupling of endothelial and neuronal cells. Nat Biotechnol *36*: 865-+.
11. Wojciak-Stothard, B., Abdul-Salam, V. B., Lao, K. H., Tsang, H., Irwin, D. C., Lisk, C., Loomis, Z., Stenmark, K. R., Edwards, J. C., Yuspa, S. H.*, et al.* (2014). Aberrant Chloride Intracellular Channel 4 Expression Contributes to Endothelial Dysfunction in Pulmonary Arterial Hypertension. Circulation *129*: 1770-1780.
12. Aman, J., van Bezu, J., Damanafshan, A., Huveneers, S., Eringa, E. C., Vogel, S. M., Groeneveld, A. B., Vonk Noordegraaf, A., van Hinsbergh, V. W., and van Nieuw Amerongen, G. P. (2012). Effective treatment of edema and endothelial barrier dysfunction with imatinib. Circulation *126*: 2728-2738.
13. Casserly, B., and Klinger, J. R. (2009). Ambrisentan for the treatment of pulmonary arterial hypertension. Drug Des Devel Ther *2*: 265-280.
14. Van der Feen, D. E., Kurakula, K., Tremblay, E., Boucherat, O., Bossers, G. P. L., Szulcek, R., Bourgeois, A., Lampron, M. C., Habbout, K., Martineau, S.*, et al.* (2019). Multicenter Preclinical Validation of BET Inhibition for the Treatment of Pulmonary Arterial Hypertension. Am J Respir Crit Care Med *200*: 910-920.
