# Supplementary material for: An organ-on-chip model of pulmonary arterial hypertension identifies a BMPR2-SOX17-prostacyclin signalling axis
Source: Commun Biol. 2022 Nov 7;5:1192. doi: 10.1038/s42003-022-04169-z (PMC9640600; doi:10.1038/s42003-022-04169-z)
Supplement: Supplementary file 3 — Description of Additional Supplementary Files [file 42003_2022_4169_MOESM3_ESM.pdf]

Description of Additional Supplementary Files

File name: Supplementary Data 1

Description: List of DEGs in HPAECs and HPASMCs in double hit HPAEC and ECFC models

File name: Supplementary Data 2

Description: BMPR2 independent genes in double hit PAH model

File name: Supplementary Data 3

Description: Pathway analysis of BMPR2 independent genes in HPAECs double hit model

File name: Supplementary Data 4

Description: List of HPAEC and HPASMC genes affected by HPAEC BMPR2 silencing

File name: Supplementary Data 5

Description: Pathway analysis of hypoxia-induced BMPR2 independent genes in HPASMCs double hit model

File name: Supplementary Data 6

Description: List of overlapping genes in normoxic ADENO HPAEC and ECFC models

File name: Supplementary Data 7

Description: List of DEGs in BMPR2-deficient ECFC and HPASMCs

File name: Supplementary Data 8

Description: Pathway analysis of hypoxia-induced genes in ECFCs in the double hit model

File name: Supplementary Data 9

Description: Pathway analysis of hypoxia-induced genes in HPASMCs in the double hit ECFC model

File name: Supplementary Data 10

Description: All DEG datasets from our models and other PAH databases

File name: Supplementary Data 11

Description: Overlapping endothelial and smooth muscle gene lists from Figure 8

File name: Supplementary Data 12

Description: Pathway analysis of common endothelial DEGs from chip PAH models and PAH datasets

File name: Supplementary Data 13

Description: pathway analysis of common SMC DEGs from chip PAH models and PAH datasets Supplementary Data

File name: Supplementary Data 14

Description: Normalised and raw protein abundances in normoxic control vs BMPR2/hypoxia group

File name: Supplementary Data 15

Description: Normalised and raw protein abundances in normoxic control vs BMPR2/hypoxia + SOX17 group

File name: Supplementary Data 16

Description: Normalised and raw protein abundances in BMPR2/hypoxia group vs BMPR2/hypoxia + SOX17 group

File name: Supplementary Data 17

Description: Source data for graphs
