# Supplementary material for: An organ-on-chip model of pulmonary arterial hypertension identifies a BMPR2-SOX17-prostacyclin signalling axis
Source: Commun Biol. 2022 Nov 7;5:1192. doi: 10.1038/s42003-022-04169-z (PMC9640600; doi:10.1038/s42003-022-04169-z)
Supplement: Supplementary file 21 — Reporting Summary [file 42003_2022_4169_MOESM21_ESM.pdf]

nature portfolio

Corresponding author(s): COMMSBIO-22-1448C
Beata Wojciak-Stothard

Last updated by author(s): Oct 25, 2022

Reporting Summary

Nature Portfolio wishes to improve the reproducibility of the work that we publish. This form provides structure for consistency and transparency in reporting. For further information on Nature Portfolio policies, see our Editorial Policies and the Editorial Policy Checklist.

Statistics

For all statistical analyses, confirm that the following items are present in the figure legend, table legend, main text, or Methods section.

n/a	Confirmed	
☐	☒	The exact sample size (*n*) for each experimental group/condition, given as a discrete number and unit of measurement
☐	☒	A statement on whether measurements were taken from distinct samples or whether the same sample was measured repeatedly
☐	☒	The statistical test(s) used AND whether they are one- or two-sided<br>*Only common tests should be described solely by name; describe more complex techniques in the Methods section.*
☒	☐	A description of all covariates tested
☐	☒	A description of any assumptions or corrections, such as tests of normality and adjustment for multiple comparisons
☐	☒	A full description of the statistical parameters including central tendency (e.g. means) or other basic estimates (e.g. regression coefficient) AND variation (e.g. standard deviation) or associated estimates of uncertainty (e.g. confidence intervals)
☐	☒	For null hypothesis testing, the test statistic (e.g. *F*, *t*, *r*) with confidence intervals, effect sizes, degrees of freedom and *P* value noted<br>*Give P values as exact values whenever suitable.*
☒	☐	For Bayesian analysis, information on the choice of priors and Markov chain Monte Carlo settings
☒	☐	For hierarchical and complex designs, identification of the appropriate level for tests and full reporting of outcomes
☒	☐	Estimates of effect sizes (e.g. Cohen's *d*, Pearson's *r*), indicating how they were calculated

Our web collection on statistics for biologists contains articles on many of the points above.

Software and code

Policy information about availability of computer code

Data collection: Leica LAS AF Lite for acquisition and analysis of confocal images, Glomax Instinct software for plate reader, Adobe Photoshop CS5 Extended for image processing

Data analysis: GraphPad Prism 7.03, Fiji-ImageJ (NIH) for image analysis,Progenesis QI for Proteomics ,UCSC Genome Browser on Human GRCh38/hg38 build ,COMSOL Multiphysics version 4.4, GSEA pathway analysis, Ingenuity Pathway Analysis, R Studio, EnhancedVolcano

For manuscripts utilizing custom algorithms or software that are central to the research but not yet described in published literature, software must be made available to editors and reviewers. We strongly encourage code deposition in a community repository (e.g. GitHub). See the Nature Portfolio guidelines for submitting code & software for further information.

Data

Policy information about availability of data

All manuscripts must include a data availability statement. This statement should provide the following information, where applicable:
- Accession codes, unique identifiers, or web links for publicly available datasets
- A description of any restrictions on data availability
- For clinical datasets or third party data, please ensure that the statement adheres to our policy

All data generated or analysed during this study are included in this published article (and its supplementary information files). Any other information is available from the corresponding author upon reasonable request. Gene datasets will be made available upon the acceptance of the manuscript.

Human research participants

Policy information about studies involving human research participants and Sex and Gender in Research.

Reporting on sex and gender	Male and female samples were used (with female predominance, according to PAH pathology) and full information regarding sex, age and treatments has been provided in the manuscript
Population characteristics	n/a
Recruitment	n/a
Ethics oversight	London Bloomsbury Research Ethics Committee (NHS Research Ethics Committee)

Note that full information on the approval of the study protocol must also be provided in the manuscript.

Field-specific reporting

Please select the one below that is the best fit for your research. If you are not sure, read the appropriate sections before making your selection.

☒ Life sciences ☐ Behavioural & social sciences ☐ Ecological, evolutionary & environmental sciences

For a reference copy of the document with all sections, see nature.com/documents/nr-reporting-summary-flat.pdf

Life sciences study design

All studies must disclose on these points even when the disclosure is negative.

Sample size	RNAseq was carried out on samples from 4-5 different donors, with material pulled from 2 technical repeats. Sample sizes for functional studies (cell permeability, apoptosis, proliferation) were based on sample sizes used in well established, published protocols
Data exclusions	no data were excluded
Replication	All observations made in functional chips were replicated. Chips that showed an imperfect alignment of the channels or were leaky, were rejected
Randomization	samples were randomly allocated
Blinding	All personnel involved in data collection and analysis were blinded to the treatment status.

Reporting for specific materials, systems and methods

We require information from authors about some types of materials, experimental systems and methods used in many studies. Here, indicate whether each material, system or method listed is relevant to your study. If you are not sure if a list item applies to your research, read the appropriate section before selecting a response.

Materials & experimental systems

n/a	Involved in the study	
☐	☒	Antibodies
☐	☒	Eukaryotic cell lines
☒	☐	Palaeontology and archaeology
☒	☐	Animals and other organisms
☒	☐	Clinical data
☐	☐	Dual use research of concern

Methods

n/a	Involved in the study	
☒	☐	ChIP-seq
☒	☐	Flow cytometry
☒	☐	MRI-based neuroimaging

Antibodies

Antibodies used	AlexaFluor488 (Anti-Rabbit) Goat Life Technologies A11034 1:300,<br>VE-Cadherin<br>(AlexaFluor 488 Conjugated) Mouse eBioscience 53-1449-42 1:100<br>α-Smooth Muscle Actin

(AlexaFluor 647 Conjugated) Mouse Abcam ab196919 1:100
Smooth muscle calponin Rabbit Abcam ab46794 1:100
Alexa fluor-546 (Anti-Rabbit) Goat Life Technologies A11010 1:100
Alexa Fluor Plus 594 (Anti-Mouse) Goat Life Technologies A32742 1:300
Alexa Fluor Plus 594 (Anti-Rabbit) Goat Life Technologies A32740 1:300
ERG (Anti-human) Rabbit Abcam ab92513 1:100
vWF (Anti-human) Rabbit Dako A0082 1:500
α-Smooth Muscle Actin (Anti-human) Mouse Dako M0851 1:1000
Anti-KCNK1 (anit-human) Rabbit Life Technologies PA541010 1:50
FITC Anti-Rabbit Goat Jackson ImmunoResearch Inc 111-095-003 1:100
TRITC Anti-Mouse Goat Sigma-Aldrich T5393 1:100
human β-actin Mouse Santa Cruz sc-47778 1:2000
human BMPR2 Mouse BD Biosciences 612292 1:1000
HRP-labelled anti-mouse Sheep GE Healthcare NA931 1:2000

Validation: validation was provided by manufacturers

Eukaryotic cell lines

Policy information about cell lines and Sex and Gender in Research

Cell line source(s)	HPAECs (PromoCell, Cat. No. C-12241) , HPASMCs (Lonza, Cat. No. CC-2581)
Authentication	authentification certificate was provided by manufacturers
Mycoplasma contamination	No further authentication has been performed. A certificate of analysis and purity is provided for each cell lot purchased from the companies.
Commonly misidentified lines (See ICLAC register)	the study did not use misidentified cell lines

Dual use research of concern

Policy information about dual use research of concern

Hazards

Could the accidental, deliberate or reckless misuse of agents or technologies generated in the work, or the application of information presented in the manuscript, pose a threat to:

No	Yes	
☒	☐	Public health
☒	☐	National security
☒	☐	Crops and/or livestock
☒	☐	Ecosystems
☒	☐	Any other significant area

Experiments of concern

Does the work involve any of these experiments of concern:

No	Yes	
☒	☐	Demonstrate how to render a vaccine ineffective
☒	☐	Confer resistance to therapeutically useful antibiotics or antiviral agents
☒	☐	Enhance the virulence of a pathogen or render a nonpathogen virulent
☒	☐	Increase transmissibility of a pathogen
☒	☐	Alter the host range of a pathogen
☒	☐	Enable evasion of diagnostic/detection modalities
☒	☐	Enable the weaponization of a biological agent or toxin
☒	☐	Any other potentially harmful combination of experiments and agents
